# Germany as a key transit hub for the emergence and spread of high pathogenicity avian influenza H5 clade 2.3.4.4b reassortants in Europe

**DOI:** 10.3389/fmicb.2026.1824729

**Published:** 2026-05-28

**Authors:** Ann Kathrin Ahrens, Christian Grund, Martin Beer, Timm C. Harder, Anne Pohlmann

**Affiliations:** Institute of Diagnostic Virology, Friedrich-Loeffler-Institut, Greifswald, Germany

**Keywords:** H5 clade 2.3.4.4b, high pathogenicity avian influenza virus, phylogeography, surveillance, viral reassortment, virus evolution, wild birds

## Abstract

High pathogenicity avian influenza (HPAI) H5 viruses of the Goose/Guangdong lineage continue to diversify through recurrent reassortment, sustaining an evolving threat to animal health and transboundary disease control in Europe. While multiple incursions of HPAI H5 clade 2.3.4.4b viruses have been documented, the regional and ecological contexts that facilitate reassortment during ongoing circulation remain insufficiently characterized. In this study, we combined nationwide avian influenza surveillance in Germany with whole-genome sequencing and Bayesian phylogeographic reconstruction to investigate virus evolution during the 2024–2025 season. Phylogenetic analyses identified multiple genetically distinct HPAI H5 clade 2.3.4.4b genotypes, including several reassortants that emerged during the observation period. The inferred evolutionary histories indicate repeated virus introductions into Germany, followed by local diversification and onward dissemination across Europe. Spatio-temporal reconstruction consistently highlighted coastal regions along the North Sea and Baltic Sea as major interfaces of viral exchange, in line with their role as convergence zones of migratory bird flyways. Rather than representing a primary source of viral lineages, phylogeographic analyses identified Germany as prominent node within the European HPAI network, consistent with a central role in virus movement and mixing. This pattern was observed across multiple genotypes, supporting a generalized role of this region in HPAI evolution. Together, these findings provide a continental-scale perspective on HPAI virus dynamics and emphasize the value of integrated surveillance approaches combining wild bird monitoring and whole-genome sequencing. From an epidemiological standpoint, expanding surveillance frameworks to include low pathogenic avian influenza viruses is likely to improve early detection of reassortment events and enhance preparedness for the emergence of novel HPAI variants.

## Introduction

High pathogenicity avian influenza (HPAI) viruses of the Goose/Guangdong (gs/GD) lineage have established sustained circulation in wild bird populations across large parts of Eurasia and beyond (Xie et al., 2023). Since their first detection in the mid-1990s, H5 viruses of this lineage have repeatedly caused large-scale outbreaks in wild birds and domestic poultry, accompanied by substantial economic losses and ongoing concerns regarding zoonotic spillover. The remarkable evolutionary success of these viruses is driven not only by the accumulation of point mutations, but also by their pronounced capacity for genetic reassortment during co-circulation with other influenza A viruses.

Pathogenicity of avian influenza viruses is primarily determined by the cleavage site motif of the hemagglutinin (HA) precursor protein. While low pathogenic avian influenza viruses (LPAIV) typically harbor a trypsin-sensitive monobasic cleavage site restricting replication to the respiratory and intestinal tract (Luczo and Spackman, 2025), HPAIV of subtypes H5 and H7 possess a multibasic cleavage site that enables systemic spread through ubiquitous furin-like proteases (Decha et al., 2008). Beyond pathogenicity, however, the segmented genome of influenza A viruses provides the mechanistic basis for reassortment, allowing the exchange of entire gene segments between co-infecting viruses and thereby generating novel genotypes with altered biological properties (Taylor et al., 2023).

In recent years, reassortment has emerged as a dominant evolutionary force shaping the diversity of gs/GD-like HPAI H5 clade 2.3.4.4b viruses in Europe. Numerous novel genotypes have been detected within short time frames, reflecting intense co-circulation and frequent genetic exchange among HPAI and LPAIV strains (Ahrens et al., 2024b; Fusaro et al., 2024; Van Borm et al., 2025). Some of these reassortant viruses have attracted particular attention due to their ability to infect mammalian hosts, including notable spillover events such as a die-off of cats in Poland in 2023 (Domanska-Blicharz et al., 2023; Rabalski et al., 2023) or infection of farmed minks and ferrets in Finland in the same year (Kareinen et al., 2024).

Despite extensive surveillance and increasingly detailed genotype cataloging, it remains poorly understood where and under which conditions reassortment events preferentially occur at a continental scale. In particular, regions located at the intersection of major migratory bird flyways may act as convergence zones where genetically diverse virus lineages co-circulate, creating opportunities for reassortment and onward dissemination. However, the role of such regions as transit and mixing zones within European HPAI transmission networks has received limited systematic investigation.

Germany has a specific ecological and epidemiological profile within Europe. Situated at the intersection of the East Atlantic and Black Sea–Mediterranean flyways and characterized by extensive coastal wetlands along the North Sea and Baltic Sea, the country is repeatedly exposed to diverse avian influenza virus lineages. This raises the hypothesis that Germany may function less as a primary source region and more as a central transit and mixing node that facilitates viral reassortment during large-scale circulation.

In this study, we combined nationwide avian influenza surveillance in Germany with whole-genome sequencing and Bayesian phylogeographic analyses to investigate the evolution and spread of HPAI H5 clade 2.3.4.4b viruses during the 2024–2025 season. Our objectives were to characterize the diversity of circulating genotypes, identify the emergence of novel reassortants, and assess the role of Germany within European transmission and reassortment networks.

## Results

### Number and local distribution of cases

**Figure 1:**
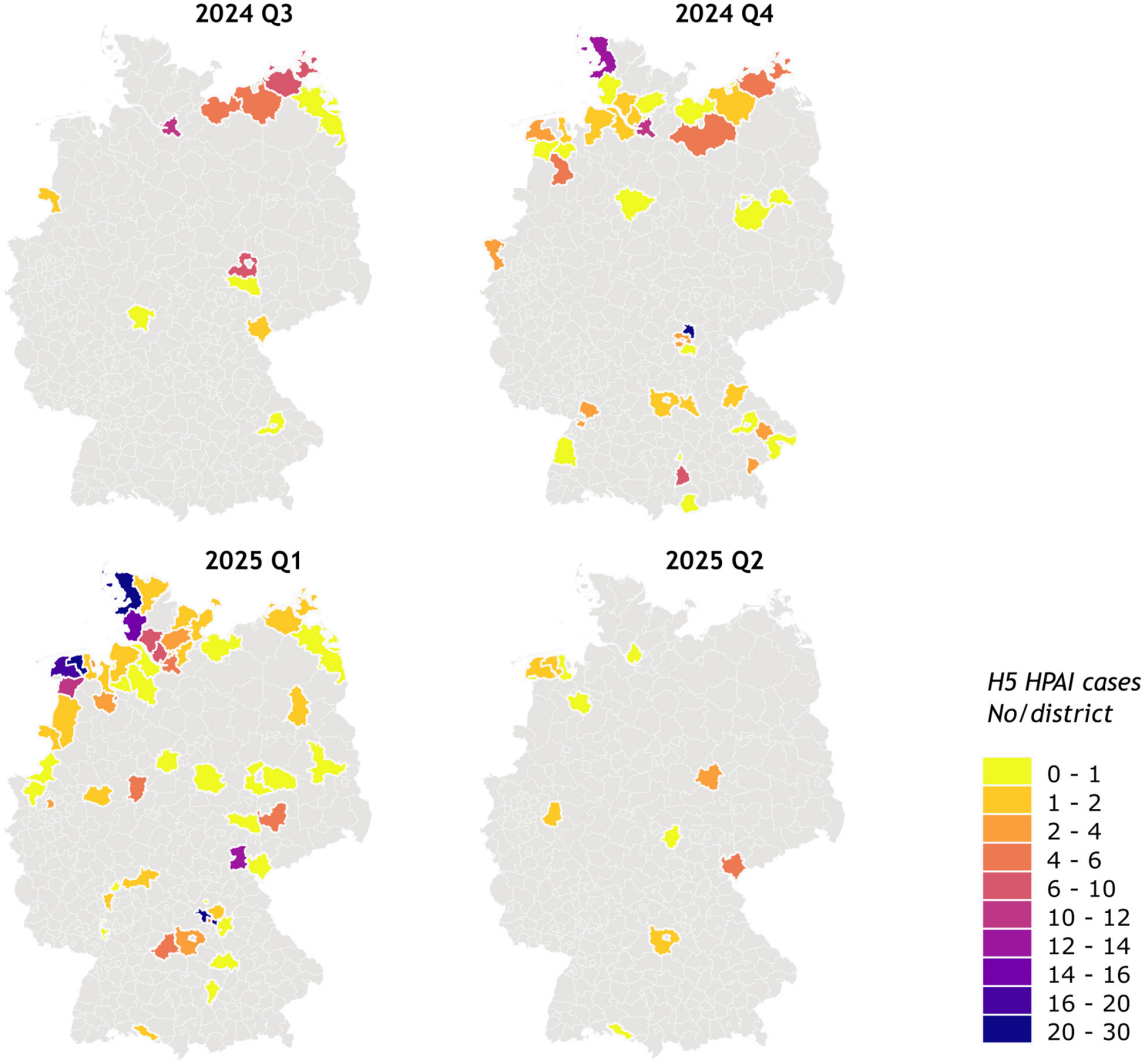
Spatial and temporal distribution of high pathogenicity avian influenza H5 cases in Germany (2024–2025). District-level distribution of confirmed high pathogenicity avian influenza virus (HPAIV) subtype H5 detections from July 2024 to July 2025. Districts are colored according to the number of detected cases per quarter. Data include wild bird detections and outbreaks in domestic poultry.

We analyzed a 12-month period from July 2024 to July 2025 in Germany, integrating nationwide data on outbreaks of high pathogenicity HPAIV subtype H5. During this period, 441 HPAI events were reported, comprising 393 detections in wild birds and 48 outbreaks in domestic poultry. Of the detections in wild birds, 258 (66%) were from birds found dead, 67 from surveillance monitoring, and 38 from samples examined for other reasons, such as carcass submissions. A further 11 birds were sampled following clinical suspicion of disease. Only 17 samples were collected from live finds, of which 14 were found alive but subsequently sacrificed due to signs of illness. Only one sample originated from active capture or hunting, for example by trapping. The district-level spatial distribution of all events is shown in Figure 1. HPAI activity was low during the summer months of 2024 but increased progressively toward the end of the year, with elevated case numbers during the winter season. The highest level of activity was observed in early 2025, followed by a gradual decline over the subsequent months. Pronounced spatial shifts in case distribution were observed over time. In the second half of 2024, detections were primarily concentrated in north-eastern Germany, particularly in Mecklenburg–Western Pomerania and the federal state of Hamburg, with additional cases reported from southern Saxony-Anhalt. During the fourth quarter of 2024, the focus shifted toward north-western Germany, including parts of Lower Saxony and Schleswig-Holstein, as well as southern regions, most notably Bavaria. In the first quarter of 2025, HPAI activity was predominantly concentrated along Germany's northern border regions, including areas bordering the Netherlands, Denmark, the Baltic Sea, and Poland. In addition, localized clusters affecting both wild birds and poultry were detected in parts of Lower Saxony and Schleswig-Holstein, as well as regional peaks in southern Thuringia and western Bavaria. During the second quarter of 2025, case numbers declined substantially, with detections occurring at lower frequency and showing a more diffuse spatial distribution across Germany.

### Host distribution

To further characterize the epidemiological patterns underlying these spatial and temporal dynamics, we next analyzed the host distribution of HPAI H5 detections.

Host distribution was analyzed for all confirmed HPAI H5 detections in wild birds and poultry. Among the 393 wild bird cases recorded during the 2024–2025 season, detections were predominantly associated with waterfowl. The majority of cases affected *Anser anser* (49.9%) and birds of the genus Cygnus (20.6%), followed by *Laridae* species (7.0%), birds of prey (3.6%), and other wild bird species (9.4%; Table 1).

**Table 1:** Comparison of the most abundant HPAIV positive tested wild bird species in 2024/2025 with comparison in the previous season 2023/2024.

Host	Case numbers	
	2023/2024 (07/23–07/24)	2024/2025 (07/24–07/25)
Cygnus	6 (1.8%)	91 (20.6%)
*Anser anser*	103 (31.1%)	220 (49.9%)
*Laridae*	77 (23.3%)	31 (7%)
Birds of prey	22 (7.5%)	14 (3.6%)
Other wild birds	87 (29.4%)	37 (9.4%)
Total no of cases	**295**	**393**

Bold values are total numbers of cases.

Compared to the previous season (July 2023–July 2024), a pronounced shift in host distribution was observed. During the 2023–2024 season, wild bird detections were more evenly distributed across host groups, with *Anser anser* accounting for 31.1% of cases, *Laridae* for 23.3%, other wild birds for 29.4%, birds of prey for 7.5%, and Cygnus species representing only a small fraction (1.8%; Table 1). In contrast, the 2024–2025 season was characterized by a marked increase in detections among Anser and Cygnus species, accompanied by a substantial decline in cases involving *Laridae*, birds of prey, and other wild bird species.

In contrast to the pronounced host shift observed in wild birds, the number of outbreaks in domestic poultry remained relatively stable between the two seasons, with no apparent change in the affected poultry host spectrum.

### Sequencing and assignment of genotypes

To assess whether the observed shifts in host distribution were associated with changes in viral genetic diversity, we next performed whole-genome sequencing and genotype assignment of selected HPAI H5 isolates.

Samples for whole-genome sequencing were selected based on geographic origin, sampling date, and viral load as determined by RT-qPCR. In total, 100 HPAI H5 genomes generated from samples collected in Germany during the 2024–2025 season were successfully sequenced and assigned to genotypes.

Genotype classification followed the nomenclature scheme proposed by the European Reference Laboratory for Avian Influenza (Fusaro et al., 2024). Overall, six H5N1 genotypes and one H5N5 genotype were identified. The dominant genotype detected during the study period was euDI, which was found in both wild birds and domestic poultry and accounted for the majority of sequenced cases. Phylogenetic analyses confirmed that genotype euDI segregates into two distinct clusters, designated euDI.1 and euDI.2, consistent with previous observations at the European level (EFSA et al., 2024, 2025). Genotypes euDI.1 and euDI.2 exhibited highly consistent distance patterns across all segments (Supplementary Figure S1), supporting their interpretation as stable genomic backbones contrasting deviations in distance structure interpreted as evidence of segment-specific evolutionary processes.

In addition to euDI, three genotypes (euI^*^, euBB, and euI) that had emerged previously elsewhere in Europe were detected sporadically in Germany. The euI^*^ genotype, representing an H5N5 variant of euI characterized by a neuraminidase stalk deletion, was detected only twice during the study period. Genotype euBB, previously associated with gull-adapted virus populations, was identified in two wild bird cases in spring 2025.

**Figure 2:**
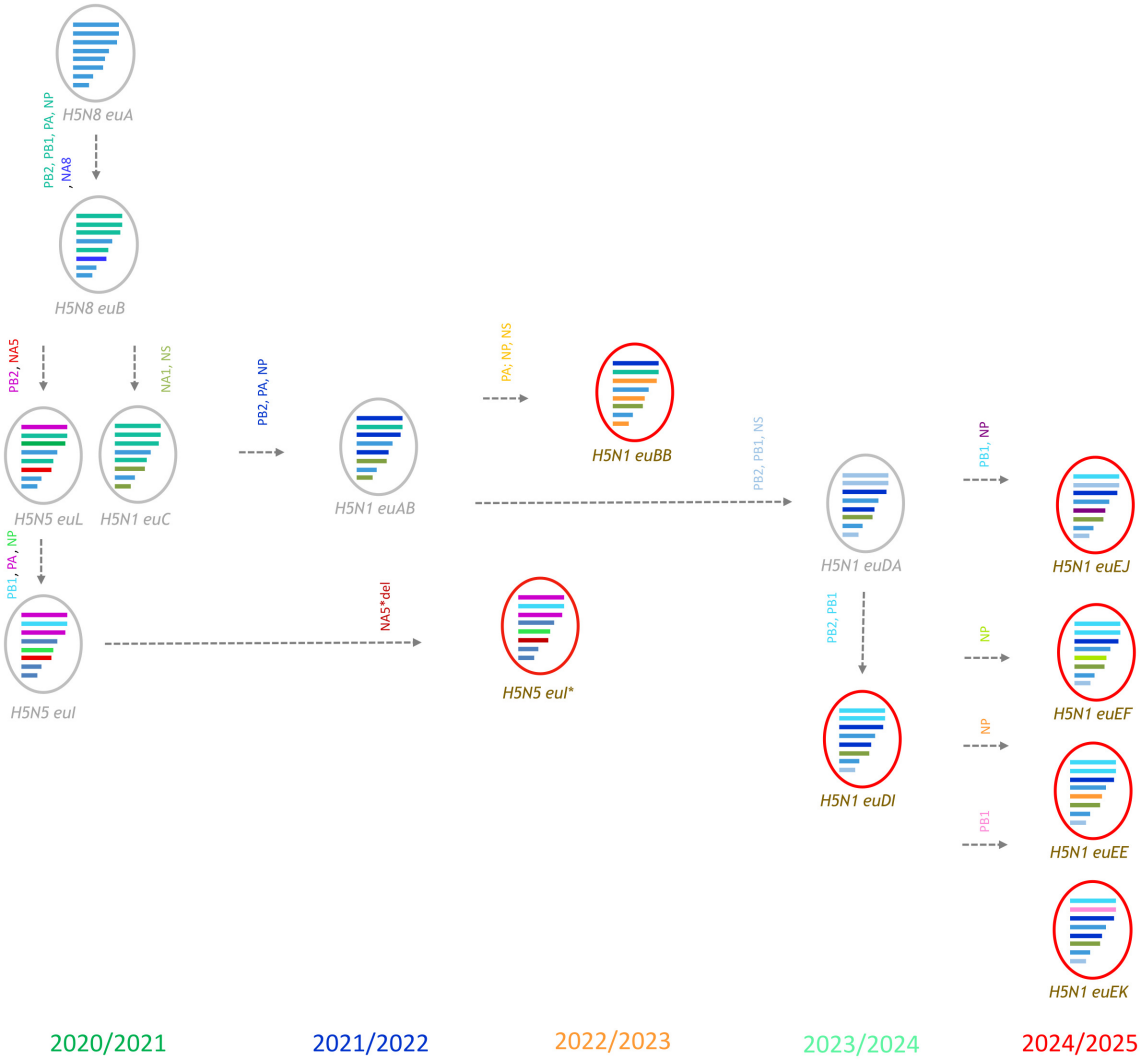
Genotype composition and reassortment patterns. Schematic representation of the genetic composition of high pathogenicity avian influenza H5 clade 2.3.4.4b genotypes detected in Germany between July 2024 and July 2025. Colored segments indicate gene segments derived from different time precursor genotypes. Genotypes detected during the study period are highlighted red, while putative precursor genotypes detected in earlier seasons are shown for reference. The season of first detection is indicated.

Notably, four novel genotypes were detected for the first time during the 2024–2025 season. Genotype euEJ was identified in Germany and is characterized by novel PB1 and NP gene segments, while the remaining genomic segments are derived from genotype euDA from the 2023–2024 season. Three additional genotypes (euEF, euEE, and euEK) were derived from the euDI backbone and contained newly acquired internal gene segments, either NP (euEF, euEE) or PB1 (euEK). Segment-wise phylogenetic gene synteny through topological comparison of the eight segments of the detected genotypes is available in Supplementary Figure S2. The genetic composition of all detected genotypes, their putative precursors, and the season of first emergence are summarized in Figure 2.

### Detection in mammalian hosts

In addition to avian hosts, three mammalian infections were identified in the dataset, all sampled in March 2025. Two cases originated from Mecklenburg-Western Pomerania (Northeast Germany), including one badger (*Mustelidae*) and one red fox (*Vulpes vulpes*), both assigned to genotype euEE. These detections occurred in close spatial and temporal proximity, suggesting a potential common infection source or a localized transmission cluster within wildlife. A third case was identified in a red fox (*Vulpes vulpes*) from Saxony (Southeast Germany) and was assigned to genotype euDI.2. Screening for known mammalian adaptation markers revealed that all three mammalian-derived genomes carried the PB2-E627K substitution. No additional hallmark mutations associated with mammalian adaptation were detected in these sequences.

### Emergence and spread of genotypes

To further investigate the temporal emergence and spatial dissemination of the dominant and newly emerged genotypes, we next performed time-scaled phylogenetic and phylogeographic analyses.

The temporal emergence and spatial dissemination of circulating HPAI H5 genotypes were investigated using time-scaled phylogenetic and phylogeographic analyses based on concatenated whole-genome sequences, as described in the Section Materials and methods. Datasets were generated for the dominant genotype euDI and for newly emerged genotypes with sufficient sequence representation collected from Germany (Supplementary Table S1) and supplemented with sequences from public databases with broad representation across countries and time (Supplementary Table S2).

Time-scaled maximum clade credibility (MCC) trees were inferred for euDI.1, euDI.2, euEF, and euEE (Supplementary Figure S3). Estimates of the most recent common ancestors (tMRCA; overview of key parameters in Supplementary Table S3) indicated an earlier emergence of euDI.1 compared to euDI.2 (euDI.1 = 2023.68, euDI.2 = 2023.98), followed by the later appearance of the euEF genotype (euEF = 2024.76). The dataset for euEE, where only limited number of sequences are available, showed greater uncertainty with a very broad 95% highest posterior density (HPD) interval (2014–2025). As this dataset is the smallest, it conveys data on a new emerging variant with limited spread.

**Figure 3:**
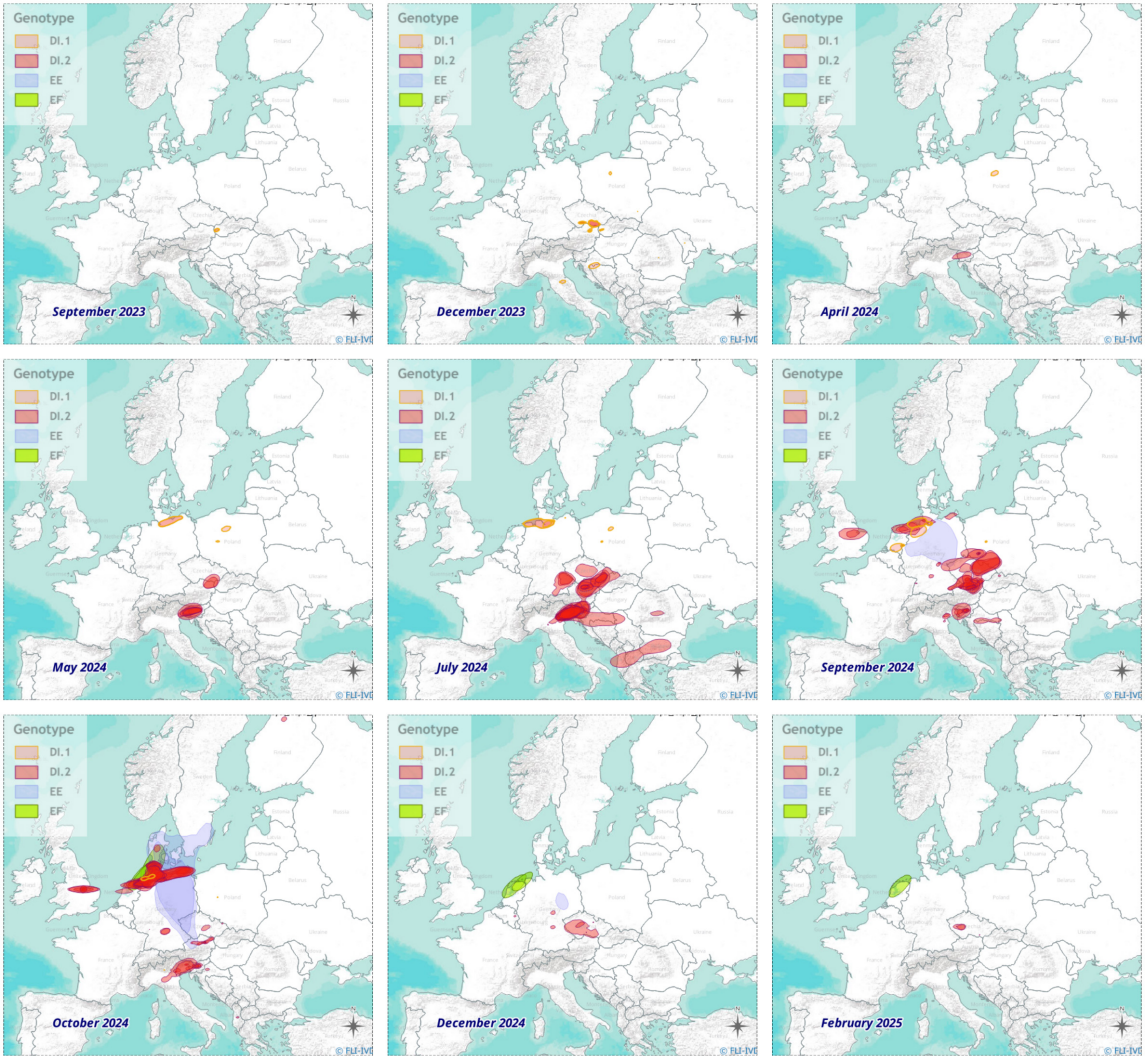
Phylogeographic reconstruction of high pathogenicity avian influenza H5 spread in Europe. Spatio-temporal phylogeographic reconstruction of high pathogenicity avian influenza H5 clade 2.3.4.4b virus spread in Europe based on concatenated whole-genome sequences collected between 2023 and 2025. Polygons represent regions of 95% highest posterior density inferred using Bayesian continuous diffusion models. Different genotypes are indicated by distinct colors. An animated visualization of the reconstruction is provided as Supplementary material.

Spatial diffusion patterns and regions of high posterior density are summarized in Figure 3, with animated reconstructions provided as Supplementary material.

Genotypes euDI.1 and euDI.2 displayed broadly similar spatial dissemination patterns but differed markedly in their timing of emergence. EuDI.1 was inferred to have emerged in eastern Europe in late 2023, followed by spread toward central and southern Europe during the subsequent winter months. The first incursions into Germany were detected in early 2024, initially in eastern regions, followed by further detections during the summer months along the Baltic Sea and North Sea coastlines. From these regions, onward spread to neighboring countries, including Denmark, the Netherlands, and the United Kingdom, was inferred.

Genotype euDI.2 emerged several months later, in mid-2024, but exhibited a comparable spatial expansion. Following its emergence, euDI.2 was detected across multiple central and northern European regions and reached coastal areas of Germany in late summer 2024. Subsequent spread along the North Sea and Baltic Sea coastlines was inferred, with detections in several neighboring countries during the following months. Over time, euDI.2 gradually replaced euDI.1 as the predominant genotype.

Genotype euEF was first detected in domestic poultry in northern Germany in late 2024. Phylogeographic reconstruction indicated a spatially restricted spread primarily associated with coastal regions, with subsequent detections in neighboring countries. Similarly, genotype euEE was first detected in early 2025 in Germany and Austria, with phylogenetic analyses suggesting an emergence toward the end of 2024. Subsequent spread was inferred toward northern Europe, particularly along the southern Baltic Sea region. Two additional novel genotypes, euEJ and euEK, were detected exclusively in Germany during the study period. Genotype euEJ was identified in early 2025 in north-western Germany, whereas genotype euEK was detected later in 2025 in western Germany, including both domestic poultry and a wild bird from the same region. Due to limited sequence numbers, no detailed phylogeographic reconstruction was performed for these genotypes.

Together, these analyses reveal distinct temporal and spatial patterns among dominant and newly emerged HPAI H5 genotypes, providing a framework for interpreting the role of Germany within European transmission networks.

## Discussion

During the 2024–2025 avian influenza season, Germany experienced extensive circulation of high pathogenicity avian influenza HPAI H5 clade 2.3.4.4b viruses in wild birds and domestic poultry, accompanied by substantial genetic diversification. By integrating nationwide surveillance data with whole-genome sequencing and phylogeographic analyses, this study provides a comprehensive view of the spatial, temporal, and evolutionary dynamics of HPAI H5 viruses in a central European setting.

A key finding of this study is the concurrent circulation of multiple genetically distinct HPAI H5 genotypes, including several reassortants that emerged during the observation period. The dominance of genotype euDI, together with the detection of additional, less prevalent genotypes, highlights the dynamic nature of HPAI evolution under conditions of sustained virus circulation. These observations are consistent with recent reports from other European regions, underscoring that reassortment has become a defining feature of the current HPAI H5 clade 2.3.4.4b epizootic.

The detection of HPAI H5 viruses in three mammalian hosts highlights the capacity of these viruses to infect beyond avian species. All mammalian-derived genomes carried the PB2-E627K substitution, a marker associated with enhanced replication in mammals. Its presence in two closely related euEE viruses from the same region suggests a shared infection source, while its occurrence in a genetically distinct euDI.2 virus indicates that this mutation can arise or be maintained across different genetic backgrounds. Despite this, we found no evidence for sustained mammalian transmission. The cases are therefore consistent with sporadic spillover events, in line with previous reports (Rabalski et al., 2023; Kareinen et al., 2024). These findings underscore the importance of continued genomic surveillance across host species.

Phylogeographic reconstruction revealed repeated virus introductions into Germany followed by local diversification and onward spread to neighboring countries. Notably, coastal regions along the North Sea and Baltic Sea repeatedly emerged as focal areas of virus circulation and dissemination. Rather than acting as a primary source of novel viral lineages, Germany appears to function predominantly as a transit and mixing node within the European HPAI transmission network. In this setting, diverse virus lineages converge, co-circulate, and reassort, facilitating the emergence of novel genotypes that subsequently disperse along major transmission routes.

The observed spatial patterns closely align with major migratory bird flyways and staging areas, supporting the notion that ecological connectivity plays a central role in shaping HPAI dynamics at a continental scale. Similar node-like functions have previously been proposed for other regions located along major flyways, suggesting that reassortment hotspots may arise where high host density, prolonged stopover periods, and repeated virus introductions coincide. Importantly, our data indicate that such regions are not necessarily sites of virus origin, but rather key interfaces where viral diversity is generated and redistributed.

Several of the newly detected genotypes displayed limited spatial spread and were restricted to specific regions or short time periods. This heterogeneity in genotype persistence and dissemination suggests that only a subset of reassortant viruses achieves sustained transmission, while many emerge transiently and disappear. Such dynamics are characteristic of influenza virus evolution and highlight the importance of dense genomic surveillance to capture these short-lived evolutionary events.

This study has several limitations. Surveillance intensity and sampling density varied across regions and host species, potentially influencing the detection frequency of specific genotypes. The reported case counts reflect detection capacity as well as true disease burden, and thus even the sequencing-effort comparison cannot fully exclude a contribution of laboratory capacity to the inferred centrality of Germany. In addition, phylogeographic inference relies on the availability of representative sequence data from multiple countries, and uneven data coverage may affect the resolution of inferred transmission pathways. Nevertheless, the consistency of observed patterns across multiple genotypes supports the robustness of the central conclusions.

From a surveillance perspective, our findings emphasize the value of integrated approaches that combine wild bird monitoring with systematic whole-genome sequencing. In particular, expanding surveillance frameworks to include low pathogenic avian influenza viruses (LPAIV) may further improve early detection of emerging variants and reassortment events, as previous studies have highlighted the role of LPAIV in viral evolution and gene segment exchange (Lu et al., 2014; Bellido-Martín et al., 2026). Such efforts are essential to strengthen preparedness and inform risk assessment in an era of sustained HPAI circulation.

Taken together, our findings identify Germany as an important mixing and transit node for high pathogenicity avian influenza viruses in Europe, highlighting how ecological connectivity and sustained circulation jointly drive reassortment and viral diversification.

## Materials and methods

RNA of influenza positive samples was extracted using the QIAamp Viral RNA Mini Kit (# 52904, Qiagen, Hilden, Germany) as previously described (Ahrens et al., 2024a; Halwe et al., 2024). Sequencing of avian influenza-positive samples was performed using an amplicon-based protocol on nanopore platforms (Oxford Nanopore Technology, Oxford, UK). Briefly, RNA was transcribed into DNA using the Superscript III One-Step and Platinum Taq kit (Thermo Fisher Scientific, Thermofischer, Waltham, MA, USA) with Influenza A specific primers, each binding to the conserved 3′ or 5′ end of all Influenza RNA segments (Pan-IVA-1F_BsmF: TATTCGTCTCAGGG-AGCRAAAGCAGG; Pan-IVA-1R_BsmR: ATATCGTCTCGTATT-AGTAGAAACAAGG). DNA amplicons were purified with Agencourt AMPure XP magnetic beads (Beckmann Coulter, Krefeld, Germany) using DNA LoBind Tubes (Eppendorf, Wesseling-Berzdorf, Germany). Quantification of nucleic acids was done with Qubit Fluorometry (Thermo Fisher Scientific, USA). Approximately 200 ng of cDNA was sequenced using a transposase-based library preparation approach with Rapid Barcoding (SQK-RBK114, Oxford Nanopore Technologies, Oxford, UK) on a PromethION P2 Solo instrument with the latest MinKNOW (Oxford Nanopore Technologies, Oxford, UK) software core (v6.5.14). High accuracy base calling of the raw data using Dorado (Oxford Nanopore Technologies, Oxford, UK) (v7.9.8, Oxford Nanopore Technologies) was followed by demultiplexing, a quality check and a trimming step to remove poor quality, adapter and short (< 20 bp) sequences. The generated data were stored in FASTQ and POD5 data formats. The bioinformatics software suite Geneious Prime (GraphPad Software LLC, version 2025.1.3, GraphPad Software, Boston, MA, USA) was used for analysis. Sequences were trimmed to remove primer sequences. Consensus sequences were obtained using an iterative map-to-reference approach with Minimap2 (vs 2.24) (Li, 2021). Reference genomes were selected from a curated collection of all HA and NA subtypes and a selection of internal gene sequences to cover all potentially circulating viral strains. Polishing of the final genome sequences and annotation was performed manually after consensus generation (threshold matching 60% of bases of total adjusted quality).

Whole-genome sequences generated in this study (Supplementary Table S1) were complemented with publicly available European HPAI H5 sequences retrieved from international databases. The dataset was curated to remove duplicates and low-quality entries. Sequences were included to achieve broad geographic representation across countries and time, while avoiding strong overrepresentation of individual regions (Supplementary Table S2) or regions with elevated case numbers (Supplementary Figure S3). Genotype assignment and detection of novel reassortants were done by inferring segment-specific phylogenetic trees for each of the eight gene segments (PB2, PB1, PA, HA, NP, NA, M, and NS). Viral genotypes were determined by the constellation of segment lineages across the genome and reassortant viruses were identified by incongruent phylogenetic clustering across segment trees (Supplementary Figure S2). For comparability study of genotype EE, EF, EJ, EK and DI.1 and DI.2 patristic distances were derived from segment-specific phylogenetic trees and assembled into pairwise distance matrices. Matrices were symmetrized and visualized as heatmaps using a fixed genotype order to preserve comparability across segments, with a shared color scale applied to all panels (Supplementary Figure S1).

Segment-specific and concatenated whole-genome multiple alignments were generated with MAFFT (v7.450) (Katoh and Standley, 2013) and subsequent maximum likelihood (ML) trees were calculated with RAxML (v8.2.1) (Stamatakis, 2014) using a GTR GAMMA model selected by use of ModelTest with rapid bootstrapping and search for the best scoring ML tree, supported by 1,000 bootstrap replicates, or alternatively with FastTree (v2.1.11) (Price et al., 2010).

Time-scaled trees of concatenated genomes of the same genotype were calculated with the BEAST X (v10.5.0) software package using a GTR GAMMA substitution model (Baele et al., 2025), an uncorrelated relaxed clock with a lognormal distribution and coalescent constant population tree models. Phylogeographic continuous trait spatial diffusion models were calculated for genotype-based sets using a Bayesian coalescent model with latitude and longitude of the sampling. Chain lengths were set to suitable iterations and convergence checked via Tracer (v1.7.1) (Rambaut et al., 2018). Time-scaled summary maximum clade credibility trees (MCC) with 10% post- burn-in posterior were created using TreeAnnotator (v1.10.4) and visualized with FigTree (V1.4.4). The robustness of the MCC trees was evaluated using 95% highest posterior density confidence intervals at each node and posterior confidence values as branch support. The spatio-temporal diffusion models were analyzed and visualized using Spread (v.1.0.7) (Bielejec et al., 2011) and QGIS (v.3.16, QGIS.org, Non-profit Association, Grüt, Switzerland).

For a comparative analysis of results excluding artifacts resulting from concatenated genomes phylogeographic analyses were repeated for EE and EF genotypes with HA segment phylogenies only and plotted back-to-back with the results of the concatenated genomes in Supplementary Figure S5. For sensitivity analysis excluding artifacts from a potential overrepresent of German sequences a downsampling strategy was applied, by randomly creating datasets with 50% reduced German samples and repeat of the phylogeographic analysis. The results were plotted back-to-back with the results of the original data set in Supplementary Figure S6.

Geographical geojson vector maps were created with open data provided by the Federal Agency for Cartography and Geodesy (http://opendatalab.de/projects/geojson-utilities/, https://gdz.bkg.bund.de). Numbers of events were summarized with open data from the German National Animal Diseases Information System (https://tsis.fli.de).

## Data Availability

The generated sequences are publicly available in the INSDC databases (https://www.insdc.org). A list of accessions, with relevant metadata, is provided as Supplementary material (Table S1). Sequences from other laboratories are listed under DOI: 10.55876/gis8.251121kr, with the acknowledgement of the originating and submitting laboratories. We acknowledge the originating and submitting laboratories for providing the sequence data.

## Supplementary material

Supplementary Figure 1 Segment-wise patristic distance heatmaps.

Supplementary Figure 2 Synteny of segments via topological comparison of the eight segments of the detected reassortants and their progenitors.

Supplementary Figure 3 Time-scaled phylogenies. Time-scaled maximum clade credibility tree of genotype [euDI.1/euDI.2/euEF/euEE] inferred from concatenated whole-genome sequences using a Bayesian relaxed molecular clock model. Branch colors correspond to sampling locations.

Supplementary Figure 4 Sequences per reported case across countries in the BEAST data set period.

Supplementary Figure 5 Sensitivity analysis of phylogeographic reconstruction of high pathogenicity avian influenza H5 genotype EE and EF spread in Europe based on concatenated whole-genome sequences and HA sequences.

Supplementary Figure 6 Downsampling analysis of phylogeographic reconstruction of high pathogenicity avian influenza H5 genotype DI.1 and DI.2 comparing original and 50% down sampled datasets for German sequences.

Supplementary Table S1 List of accession numbers of generated sequences.

Supplementary Table S2 Overview of sequence counts per country and per genotype for datasets DI.1, DI.2, EF, and EE included in the BEAST analyses.

Supplementary Table S3 Estimates of time to the most recent common ancestor (tMRCA) and substitution rates inferred using BEAST for datasets DI.1, DI.2, EF, and EE.

Supplementary video 1 Animated version of 3.
