# Supplementary material for: Germany as a key transit hub for the emergence and spread of high pathogenicity avian influenza H5 clade 2.3.4.4b reassortants in Europe
Source: Front Microbiol. 2026 May 28;17:1824729. doi: 10.3389/fmicb.2026.1824729 (PMC13253684; doi:10.3389/fmicb.2026.1824729)
Supplement: Supplementary Figure 1 — Segment-wise patristic distance heatmaps. [file Image_1.pdf]

Supplement for:

Germany as a key transit hub for the emergence and spread of high pathogenicity avian influenza H5 clade 2.3.4.4b reassortants in Europe

Ann Kathrin Ahrens, Christian Grund, Martin Beer Timm C. Harder, and Anne Pohlmann

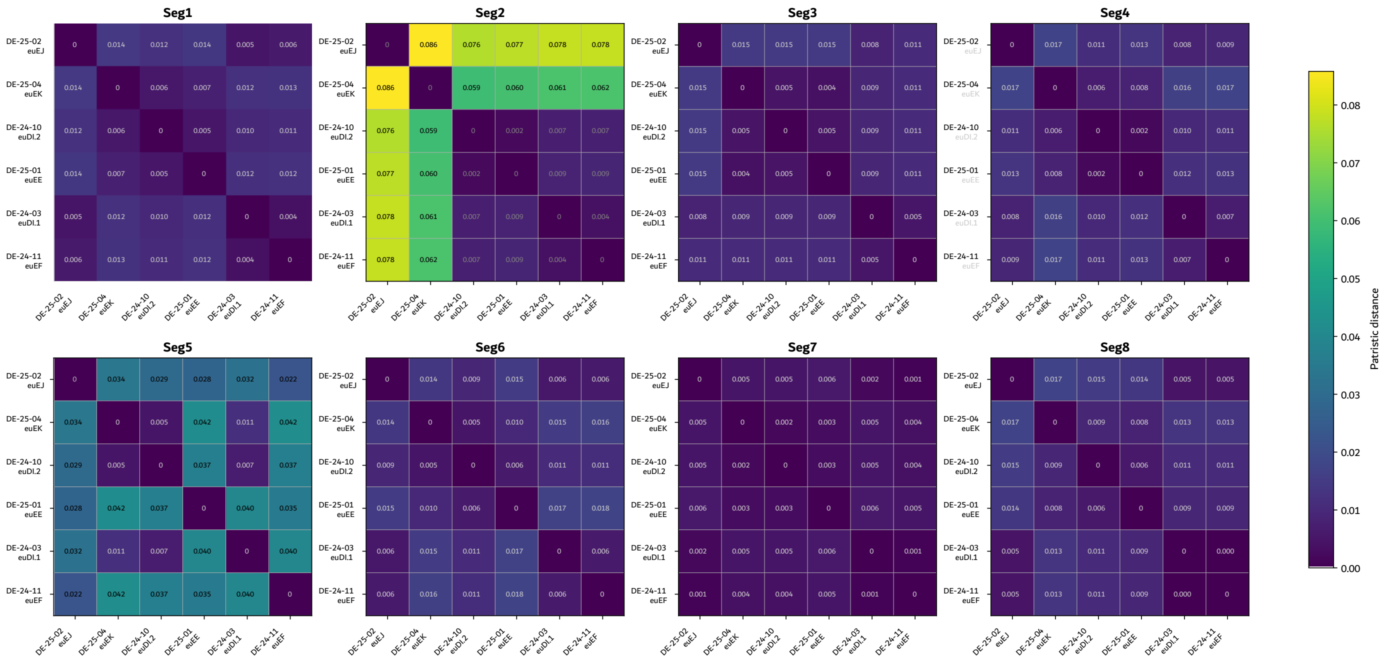

Figure S1: Segment-wise patristic distance heatmaps highlighting genomic constellations of H5N1 genotypes EE, EF, EJ, EK and DI.1, DI.2. Pairwise patristic distances were calculated across all eight genome segments and visualized as heatmaps using a fixed genotype order. Each panel represents one segment (Seg1–Seg8), with color intensity proportional to genetic distance.

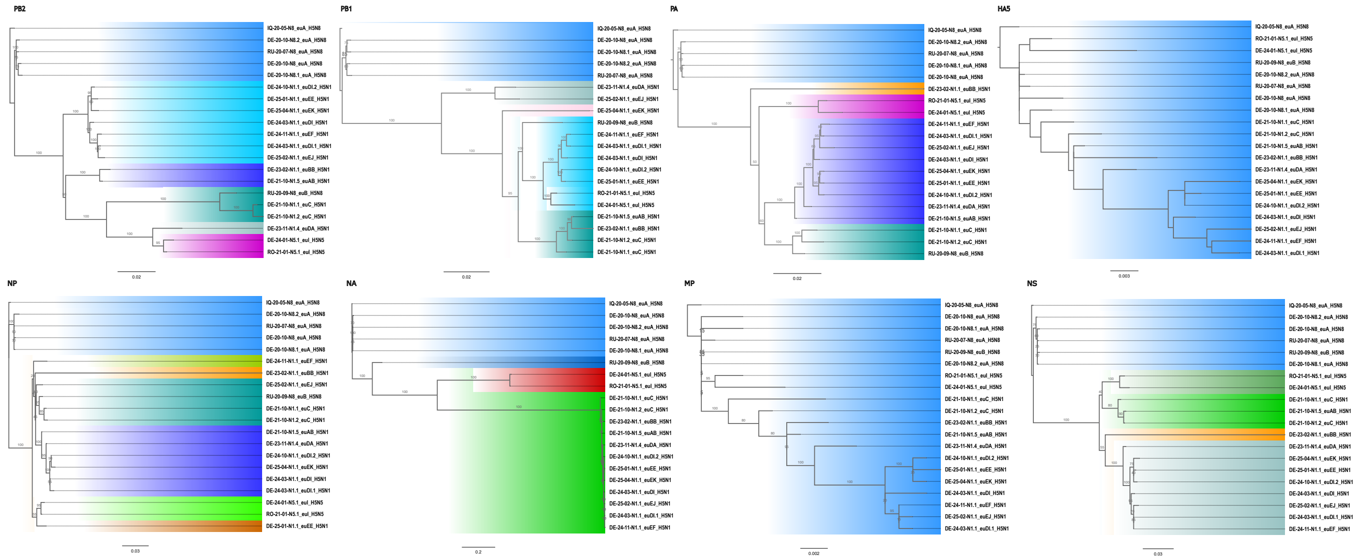

Figure S2: Synteny of segments analyzed through a topological comparison of the eight segments (PB2, PB1, PA, HA, NP, NA, MP and NS) and their respective maximum likelihood (ML) trees, obtained from all the reassortants detected in the study and their progenitors. Clusters are shown in colours. Different colours indicate discordance.

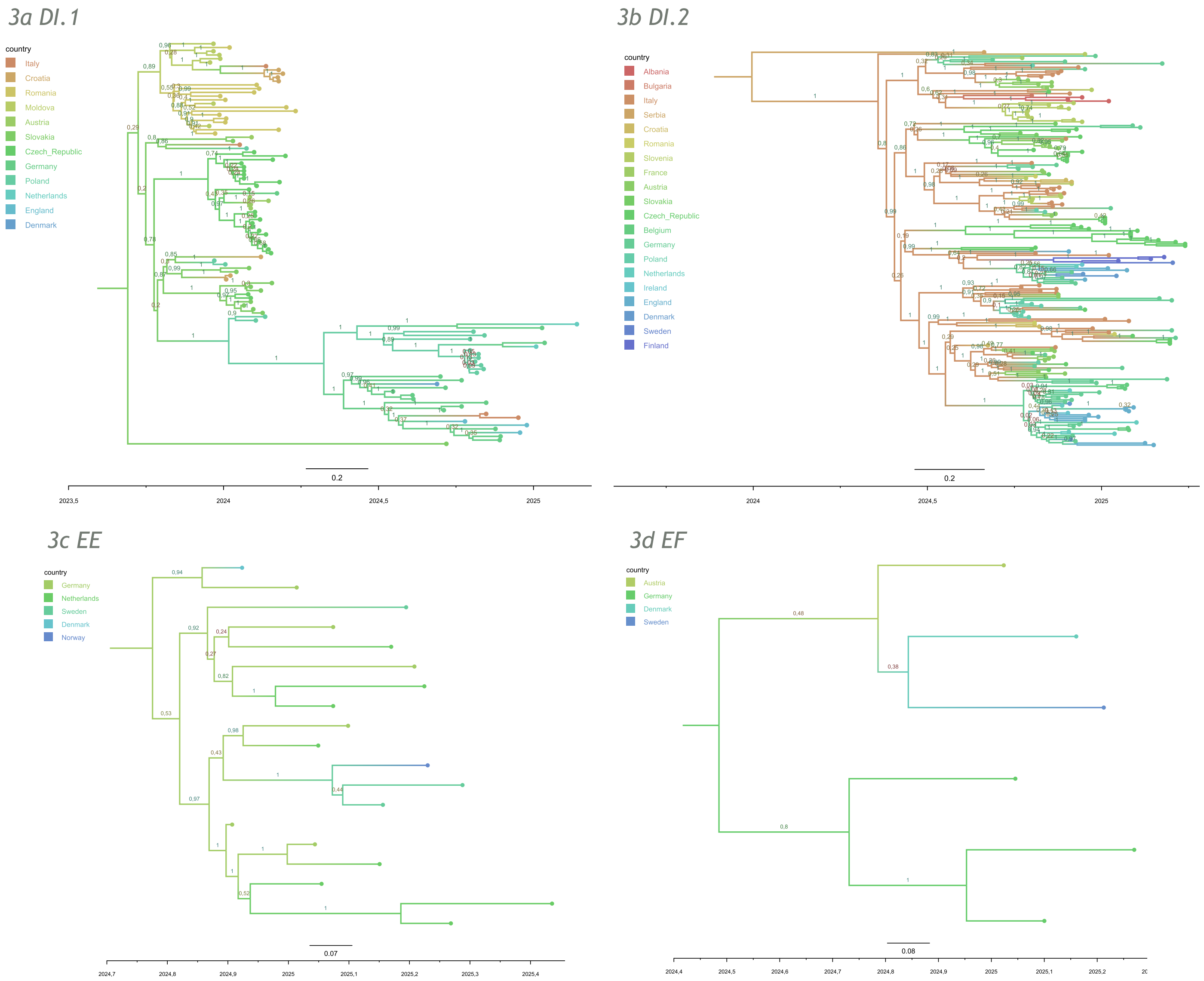

Figure S3: Time-scaled maximum clade credibility (MCC) phylogeny of concatenated H5N1 highly pathogenic avian influenza (HPAI) viral genomes of four different genotypes collected from European countries. The colours of the countries are ordered from red to blue from south to north. Posterior probabilities are given at the branch level. Figure S3a HPAI genomes of genotype DI.1; Figure S3b genotype DI.2; Figure S3c HPAI genomes of genotype EE; Figure S3d HPAI genomes of genotype EF.

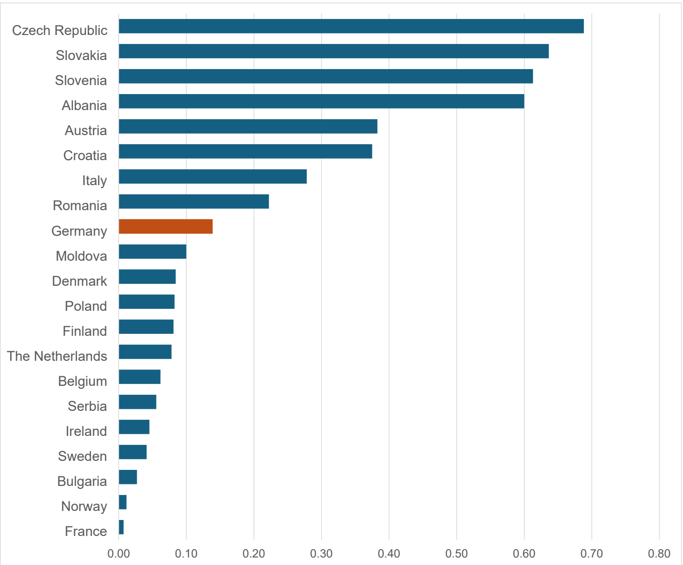

Figure S4: Sequences per reported case across countries in the BEAST data set period (12/2023-06/2025, sorted by value. Germany is marked in red. Number of events in Europe were summarized with open data from WAHIS: World Animal Health Information System.

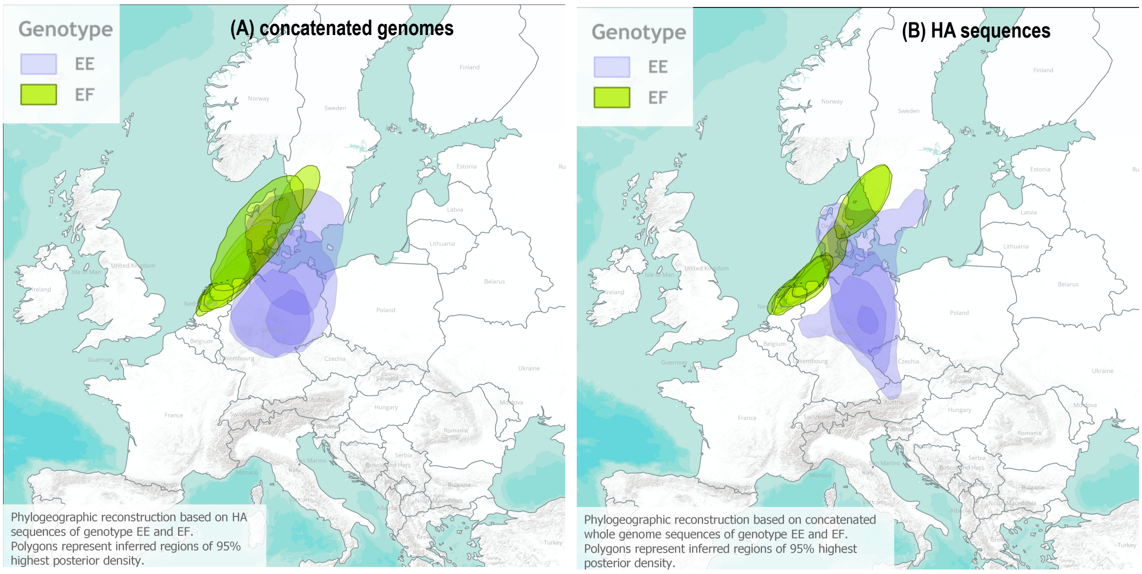

Figure S5: Sensitivity analysis of phylogeographic reconstruction of high pathogenicity avian influenza H5 spread in Europe based on (A) concatenated whole-genome sequences and (B) HA sequences collected between 2023 and 2025. Polygons represent regions of 95% highest posterior density inferred using Bayesian continuous diffusion models. Different genotypes are indicated by distinct colors.

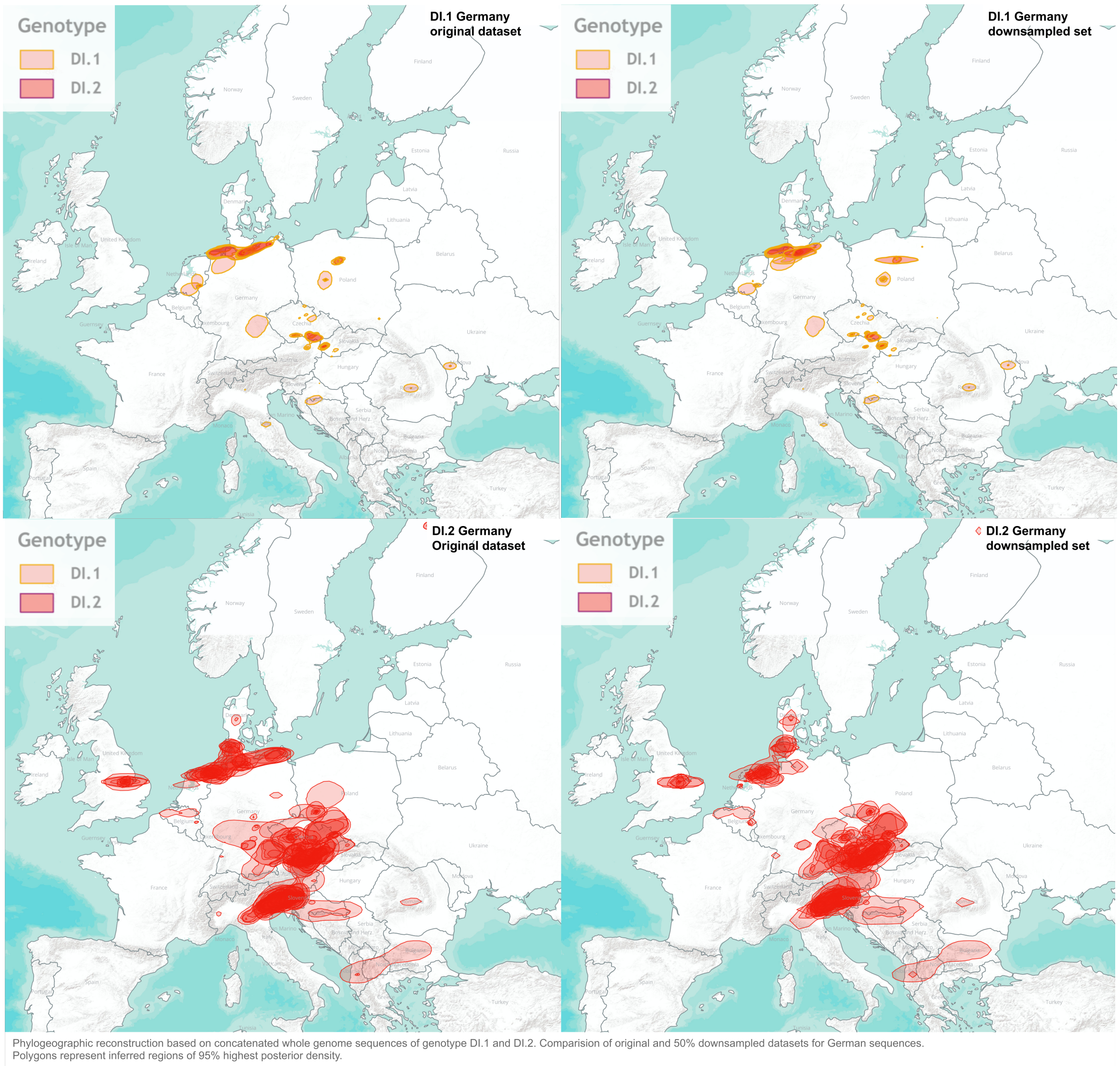

Figure S6: Downsampling analysis of phylogeographic reconstruction of high pathogenicity avian influenza H5 genotype DI.1 and DI.2 spread in Europe based on concatenated whole-genome sequences collected between 2023 and 2025. Comparison of original and 50% down sampled dataset for German sequences. Polygons represent regions of 95% highest posterior density inferred using Bayesian continuous diffusion models.
