# Supplementary material for: Germany as a key transit hub for the emergence and spread of high pathogenicity avian influenza H5 clade 2.3.4.4b reassortants in Europe
Source: Front Microbiol. 2026 May 28;17:1824729. doi: 10.3389/fmicb.2026.1824729 (PMC13253684; doi:10.3389/fmicb.2026.1824729)
Supplement: Supplementary Table S1 — List of accession numbers of generated sequences. [file Table_1.pdf]

Supplement for:

Germany as a key transit hub for the emergence and spread of high pathogenicity avian influenza H5 clade 2.3.4.4b reassortants in Europe

Ann Kathrin Ahrens, Christian Grund, Martin Beer, Timm C. Harder, and Anne Pohlmann

Table S1 Accession number and metadata of generated sequences. Data are publicly available in GenBank

Accession	Type	Isolate	Geo_Location	Collection_Date	Host
**PX671734**	H5N1	2025AI02847	Germany-NI	30.05.2025	Morus bassanus
**PX671735**	H5N1	2025AI02847	Germany-NI	30.05.2025	Morus bassanus
**PX671736**	H5N1	2025AI02847	Germany-NI	30.05.2025	Morus bassanus
**PX671737**	H5N1	2025AI02847	Germany-NI	30.05.2025	Morus bassanus
**PX671738**	H5N1	2025AI02847	Germany-NI	30.05.2025	Morus bassanus
**PX671739**	H5N1	2025AI02847	Germany-NI	30.05.2025	Morus bassanus
**PX671765**	H5N1	2024AI04006	Germany-MV	11.08.2024	Anas platyrhynchos
**PX671766**	H5N1	2024AI04006	Germany-MV	11.08.2024	Anas platyrhynchos
**PX671767**	H5N1	2024AI04006	Germany-MV	11.08.2024	Anas platyrhynchos
**PX671768**	H5N1	2024AI04006	Germany-MV	11.08.2024	Anas platyrhynchos
**PX671769**	H5N1	2024AI04006	Germany-MV	11.08.2024	Anas platyrhynchos
**PX671770**	H5N1	2024AI04006	Germany-MV	11.08.2024	Anas platyrhynchos
**PX671771**	H5N1	2024AI04006	Germany-MV	11.08.2024	Anas platyrhynchos
**PX671772**	H5N1	2024AI04006	Germany-MV	11.08.2024	Anas platyrhynchos
**PV738266**	H5N1	2025AI02204	Germany-TH	28.02.2025	Anatidae
**PV738267**	H5N1	2025AI02204	Germany-TH	28.02.2025	Anatidae
**PV738268**	H5N1	2025AI02204	Germany-TH	28.02.2025	Anatidae
**PV738269**	H5N1	2025AI02204	Germany-TH	28.02.2025	Anatidae
**PV738270**	H5N1	2025AI02204	Germany-TH	28.02.2025	Anatidae
**PV738271**	H5N1	2025AI02204	Germany-TH	28.02.2025	Anatidae
**PV738272**	H5N1	2025AI02204	Germany-TH	28.02.2025	Anatidae
**PV738273**	H5N1	2025AI02204	Germany-TH	28.02.2025	Anatidae
**PV738274**	H5N1	2025AI02371	Germany-MV	28.03.2025	Mustelidae
**PV738275**	H5N1	2025AI02371	Germany-MV	28.03.2025	Mustelidae
**PV738276**	H5N1	2025AI02371	Germany-MV	28.03.2025	Mustelidae
**PV738277**	H5N1	2025AI02371	Germany-MV	28.03.2025	Mustelidae
**PV738278**	H5N1	2025AI02371	Germany-MV	28.03.2025	Mustelidae
**PV738279**	H5N1	2025AI02371	Germany-MV	28.03.2025	Mustelidae
**PV738280**	H5N1	2025AI02371	Germany-MV	28.03.2025	Mustelidae
**PV738281**	H5N1	2025AI02371	Germany-MV	28.03.2025	Mustelidae
**PV738282**	H5N1	2025AI02376	Germany-SN	31.03.2025	Anatidae
**PV738283**	H5N1	2025AI02376	Germany-SN	31.03.2025	Anatidae
**PV738284**	H5N1	2025AI02376	Germany-SN	31.03.2025	Anatidae

**PV738285**	H5N1	2025AI02376	Germany-SN	31.03.2025	Anatidae
**PV738286**	H5N1	2025AI02376	Germany-SN	31.03.2025	Anatidae
**PV738287**	H5N1	2025AI02376	Germany-SN	31.03.2025	Anatidae
**PV738288**	H5N1	2025AI02376	Germany-SN	31.03.2025	Anatidae
**PV738289**	H5N1	2025AI02376	Germany-SN	31.03.2025	Anatidae
**PV738290**	H5N1	2025AI02377	Germany-BY	19.03.2025	Cygnus olor
**PV738291**	H5N1	2025AI02377	Germany-BY	19.03.2025	Cygnus olor
**PV738292**	H5N1	2025AI02377	Germany-BY	19.03.2025	Cygnus olor
**PV738293**	H5N1	2025AI02377	Germany-BY	19.03.2025	Cygnus olor
**PV738294**	H5N1	2025AI02377	Germany-BY	19.03.2025	Cygnus olor
**PV738295**	H5N1	2025AI02377	Germany-BY	19.03.2025	Cygnus olor
**PV738296**	H5N1	2025AI02377	Germany-BY	19.03.2025	Cygnus olor
**PV738297**	H5N1	2025AI02377	Germany-BY	19.03.2025	Cygnus olor
**PV738298**	H5N1	2025AI02379	Germany-SH	18.03.2025	Larus marinus
**PV738299**	H5N1	2025AI02379	Germany-SH	18.03.2025	Larus marinus
**PV738300**	H5N1	2025AI02379	Germany-SH	18.03.2025	Larus marinus
**PV738301**	H5N1	2025AI02379	Germany-SH	18.03.2025	Larus marinus
**PV738302**	H5N1	2025AI02379	Germany-SH	18.03.2025	Larus marinus
**PV738303**	H5N1	2025AI02379	Germany-SH	18.03.2025	Larus marinus
**PV738304**	H5N1	2025AI02379	Germany-SH	18.03.2025	Larus marinus
**PV738305**	H5N1	2025AI02379	Germany-SH	18.03.2025	Larus marinus
**PV738306**	H5N1	2025AI02672	Germany-NI	28.03.2025	Branta leucopsis
**PV738307**	H5N1	2025AI02672	Germany-NI	28.03.2025	Branta leucopsis
**PV738308**	H5N1	2025AI02672	Germany-NI	28.03.2025	Branta leucopsis
**PV738309**	H5N1	2025AI02672	Germany-NI	28.03.2025	Branta leucopsis
**PV738310**	H5N1	2025AI02672	Germany-NI	28.03.2025	Branta leucopsis
**PV738311**	H5N1	2025AI02672	Germany-NI	28.03.2025	Branta leucopsis
**PV738312**	H5N1	2025AI02672	Germany-NI	28.03.2025	Branta leucopsis
**PV738313**	H5N1	2025AI02672	Germany-NI	28.03.2025	Branta leucopsis
**PV738314**	H5N1	2025AI02677	Germany-ST	10.04.2025	Aves
**PV738315**	H5N1	2025AI02677	Germany-ST	10.04.2025	Aves
**PV738316**	H5N1	2025AI02677	Germany-ST	10.04.2025	Aves
**PV738317**	H5N1	2025AI02677	Germany-ST	10.04.2025	Aves
**PV738318**	H5N1	2025AI02677	Germany-ST	10.04.2025	Aves
**PV738319**	H5N1	2025AI02677	Germany-ST	10.04.2025	Aves
**PV738320**	H5N1	2025AI02677	Germany-ST	10.04.2025	Aves
**PV738321**	H5N1	2025AI02677	Germany-ST	10.04.2025	Aves
**PV738322**	H5N1	2025AI02679	Germany-NW	11.04.2025	Gallus gallus
**PV738323**	H5N1	2025AI02679	Germany-NW	11.04.2025	Gallus gallus
**PV738324**	H5N1	2025AI02679	Germany-NW	11.04.2025	Gallus gallus
**PV738325**	H5N1	2025AI02679	Germany-NW	11.04.2025	Gallus gallus
**PV738326**	H5N1	2025AI02679	Germany-NW	11.04.2025	Gallus gallus
**PV738327**	H5N1	2025AI02679	Germany-NW	11.04.2025	Gallus gallus
**PV738328**	H5N1	2025AI02679	Germany-NW	11.04.2025	Gallus gallus
**PV738329**	H5N1	2025AI02679	Germany-NW	11.04.2025	Gallus gallus

**PV738330**	H5N1	2025AI02681	Germany-NW	11.04.2025	Gallus gallus
**PV738331**	H5N1	2025AI02681	Germany-NW	11.04.2025	Gallus gallus
**PV738332**	H5N1	2025AI02681	Germany-NW	11.04.2025	Gallus gallus
**PV738333**	H5N1	2025AI02681	Germany-NW	11.04.2025	Gallus gallus
**PV738334**	H5N1	2025AI02681	Germany-NW	11.04.2025	Gallus gallus
**PV738335**	H5N1	2025AI02681	Germany-NW	11.04.2025	Gallus gallus
**PV738336**	H5N1	2025AI02681	Germany-NW	11.04.2025	Gallus gallus
**PV738337**	H5N1	2025AI02681	Germany-NW	11.04.2025	Gallus gallus
**PV738338**	H5N1	2025AI02682	Germany-NW	11.04.2025	Gallus gallus
**PV738339**	H5N1	2025AI02682	Germany-NW	11.04.2025	Gallus gallus
**PV738340**	H5N1	2025AI02682	Germany-NW	11.04.2025	Gallus gallus
**PV738341**	H5N1	2025AI02682	Germany-NW	11.04.2025	Gallus gallus
**PV738342**	H5N1	2025AI02682	Germany-NW	11.04.2025	Gallus gallus
**PV738343**	H5N1	2025AI02682	Germany-NW	11.04.2025	Gallus gallus
**PV738344**	H5N1	2025AI02684	Germany-NW	11.04.2025	Gallus gallus
**PV738345**	H5N1	2025AI02684	Germany-NW	11.04.2025	Gallus gallus
**PV738346**	H5N1	2025AI02684	Germany-NW	11.04.2025	Gallus gallus
**PV738347**	H5N1	2025AI02684	Germany-NW	11.04.2025	Gallus gallus
**PV738348**	H5N1	2025AI02684	Germany-NW	11.04.2025	Gallus gallus
**PV738349**	H5N1	2025AI02684	Germany-NW	11.04.2025	Gallus gallus
**PV738350**	H5N1	2025AI02684	Germany-NW	11.04.2025	Gallus gallus
**PV738351**	H5N1	2025AI02684	Germany-NW	11.04.2025	Gallus gallus
**PV738352**	H5N1	2025AI02686	Germany-NW	10.04.2025	Anas platyrhynchos
**PV738353**	H5N1	2025AI02686	Germany-NW	10.04.2025	Anas platyrhynchos
**PV738354**	H5N1	2025AI02686	Germany-NW	10.04.2025	Anas platyrhynchos
**PV738355**	H5N1	2025AI02686	Germany-NW	10.04.2025	Anas platyrhynchos
**PV738356**	H5N1	2025AI02686	Germany-NW	10.04.2025	Anas platyrhynchos
**PV738357**	H5N1	2025AI02686	Germany-NW	10.04.2025	Anas platyrhynchos
**PV738358**	H5N1	2025AI02686	Germany-NW	10.04.2025	Anas platyrhynchos
**PV738359**	H5N1	2025AI02687	Germany-BW	08.04.2025	Chroicocephalus ridibundus
**PV738360**	H5N1	2025AI02687	Germany-BW	08.04.2025	Chroicocephalus ridibundus
**PV738361**	H5N1	2025AI02687	Germany-BW	08.04.2025	Chroicocephalus ridibundus
**PV738362**	H5N1	2025AI02687	Germany-BW	08.04.2025	Chroicocephalus ridibundus
**PV738363**	H5N1	2025AI02687	Germany-BW	08.04.2025	Chroicocephalus ridibundus
**PV738364**	H5N1	2025AI02687	Germany-BW	08.04.2025	Chroicocephalus ridibundus
**PV738365**	H5N1	2025AI02687	Germany-BW	08.04.2025	Chroicocephalus ridibundus
**PV738366**	H5N1	2025AI02687	Germany-BW	08.04.2025	Chroicocephalus ridibundus
**PV738367**	H5N1	2025AI02691	Germany-NI	14.04.2025	Gallus gallus
**PV738368**	H5N1	2025AI02691	Germany-NI	14.04.2025	Gallus gallus
**PV738369**	H5N1	2025AI02691	Germany-NI	14.04.2025	Gallus gallus
**PV738370**	H5N1	2025AI02691	Germany-NI	14.04.2025	Gallus gallus
**PV738371**	H5N1	2025AI02691	Germany-NI	14.04.2025	Gallus gallus
**PV738372**	H5N1	2025AI02691	Germany-NI	14.04.2025	Gallus gallus
**PV738373**	H5N1	2025AI02691	Germany-NI	14.04.2025	Gallus gallus
**PV738374**	H5N1	2025AI02691	Germany-NI	14.04.2025	Gallus gallus

**PV738375**	H5N1	2025AI02374	Germany-TH	21.03.2025	Buteo buteo
**PV738376**	H5N1	2025AI02374	Germany-TH	21.03.2025	Buteo buteo
**PV738377**	H5N1	2025AI02374	Germany-TH	21.03.2025	Buteo buteo
**PV738378**	H5N1	2025AI02374	Germany-TH	21.03.2025	Buteo buteo
**PV738379**	H5N1	2025AI02374	Germany-TH	21.03.2025	Buteo buteo
**PV738380**	H5N1	2025AI02374	Germany-TH	21.03.2025	Buteo buteo
**PV738381**	H5N1	2025AI02374	Germany-TH	21.03.2025	Buteo buteo
**PV738382**	H5N1	2025AI02374	Germany-TH	21.03.2025	Buteo buteo
**PV738383**	H5N1	2025AI02194	Germany-HE	07.03.2025	Alopochen aegyptiaca
**PV738384**	H5N1	2025AI02194	Germany-HE	07.03.2025	Alopochen aegyptiaca
**PV738385**	H5N1	2025AI02194	Germany-HE	07.03.2025	Alopochen aegyptiaca
**PV738386**	H5N1	2025AI02194	Germany-HE	07.03.2025	Alopochen aegyptiaca
**PV738387**	H5N1	2025AI02194	Germany-HE	07.03.2025	Alopochen aegyptiaca
**PV738388**	H5N1	2025AI02194	Germany-HE	07.03.2025	Alopochen aegyptiaca
**PV738389**	H5N1	2025AI02194	Germany-HE	07.03.2025	Alopochen aegyptiaca
**PV738390**	H5N1	2025AI02194	Germany-HE	07.03.2025	Alopochen aegyptiaca
**PV738391**	H5N1	2025AI02099	Germany-BY	12.03.2025	Meleagris gallopavo
**PV738392**	H5N1	2025AI02099	Germany-BY	12.03.2025	Meleagris gallopavo
**PV738393**	H5N1	2025AI02099	Germany-BY	12.03.2025	Meleagris gallopavo
**PV738394**	H5N1	2025AI02099	Germany-BY	12.03.2025	Meleagris gallopavo
**PV738395**	H5N1	2025AI02099	Germany-BY	12.03.2025	Meleagris gallopavo
**PV738396**	H5N1	2025AI02099	Germany-BY	12.03.2025	Meleagris gallopavo
**PV738397**	H5N1	2025AI02099	Germany-BY	12.03.2025	Meleagris gallopavo
**PV738398**	H5N1	2025AI02099	Germany-BY	12.03.2025	Meleagris gallopavo
**PV738399**	H5N1	2025AI01378	Germany-BB	17.02.2025	Gallus gallus
**PV738400**	H5N1	2025AI01378	Germany-BB	17.02.2025	Gallus gallus
**PV738401**	H5N1	2025AI01378	Germany-BB	17.02.2025	Gallus gallus
**PV738402**	H5N1	2025AI01378	Germany-BB	17.02.2025	Gallus gallus
**PV738403**	H5N1	2025AI01378	Germany-BB	17.02.2025	Gallus gallus
**PV738404**	H5N1	2025AI01378	Germany-BB	17.02.2025	Gallus gallus
**PV738405**	H5N1	2025AI01378	Germany-BB	17.02.2025	Gallus gallus
**PV738406**	H5N1	2025AI01378	Germany-BB	17.02.2025	Gallus gallus
**PV738407**	H5N1	2025AI02195	Germany-BB	06.03.2025	Gallus gallus
**PV738408**	H5N1	2025AI02195	Germany-BB	06.03.2025	Gallus gallus
**PV738409**	H5N1	2025AI02195	Germany-BB	06.03.2025	Gallus gallus
**PV738410**	H5N1	2025AI02195	Germany-BB	06.03.2025	Gallus gallus
**PV738411**	H5N1	2025AI02195	Germany-BB	06.03.2025	Gallus gallus
**PV738412**	H5N1	2025AI02195	Germany-BB	06.03.2025	Gallus gallus
**PV738413**	H5N1	2025AI02195	Germany-BB	06.03.2025	Gallus gallus
**PV738414**	H5N1	2025AI02195	Germany-BB	06.03.2025	Gallus gallus
**PV738415**	H5N1	2025AI01384	Germany-NI	22.02.2025	Gallus gallus
**PV738416**	H5N1	2025AI01384	Germany-NI	22.02.2025	Gallus gallus
**PV738417**	H5N1	2025AI01384	Germany-NI	22.02.2025	Gallus gallus
**PV738418**	H5N1	2025AI01384	Germany-NI	22.02.2025	Gallus gallus
**PV738419**	H5N1	2025AI01384	Germany-NI	22.02.2025	Gallus gallus

**PV738420**	H5N1	2025AI01384	Germany-NI	22.02.2025	Gallus gallus
**PV738421**	H5N1	2025AI01384	Germany-NI	22.02.2025	Gallus gallus
**PV738422**	H5N1	2025AI01384	Germany-NI	22.02.2025	Gallus gallus
**PV738423**	H5N1	2025AI02683	Germany-NW	11.04.2025	Gallus gallus
**PV738424**	H5N1	2025AI02683	Germany-NW	11.04.2025	Gallus gallus
**PV738425**	H5N1	2025AI02683	Germany-NW	11.04.2025	Gallus gallus
**PV738426**	H5N1	2025AI02683	Germany-NW	11.04.2025	Gallus gallus
**PV738427**	H5N1	2025AI02683	Germany-NW	11.04.2025	Gallus gallus
**PV738428**	H5N1	2025AI02683	Germany-NW	11.04.2025	Gallus gallus
**PV738429**	H5N1	2025AI02683	Germany-NW	11.04.2025	Gallus gallus
**PV738430**	H5N1	2025AI02683	Germany-NW	11.04.2025	Gallus gallus
**PV738431**	H5N1	2025AI02685	Germany-NW	11.04.2025	Gallus gallus
**PV738432**	H5N1	2025AI02685	Germany-NW	11.04.2025	Gallus gallus
**PV738433**	H5N1	2025AI02685	Germany-NW	11.04.2025	Gallus gallus
**PV738434**	H5N1	2025AI02685	Germany-NW	11.04.2025	Gallus gallus
**PV738435**	H5N1	2025AI02685	Germany-NW	11.04.2025	Gallus gallus
**PV738436**	H5N1	2025AI02685	Germany-NW	11.04.2025	Gallus gallus
**PV738437**	H5N1	2025AI02685	Germany-NW	11.04.2025	Gallus gallus
**PV738438**	H5N1	2025AI02685	Germany-NW	11.04.2025	Gallus gallus
**PV738439**	H5N1	2025AI02367	Germany-ST	26.03.2025	Gallus gallus
**PV738440**	H5N1	2025AI02367	Germany-ST	26.03.2025	Gallus gallus
**PV738441**	H5N1	2025AI02367	Germany-ST	26.03.2025	Gallus gallus
**PV738442**	H5N1	2025AI02367	Germany-ST	26.03.2025	Gallus gallus
**PV738443**	H5N1	2025AI02367	Germany-ST	26.03.2025	Gallus gallus
**PV738444**	H5N1	2025AI02367	Germany-ST	26.03.2025	Gallus gallus
**PV738445**	H5N1	2025AI02367	Germany-ST	26.03.2025	Gallus gallus
**PV738446**	H5N1	2025AI02367	Germany-ST	26.03.2025	Gallus gallus
**PV738447**	H5N1	2025AI02248	Germany-SN	17.03.2025	Canidae
**PV738448**	H5N1	2025AI02248	Germany-SN	17.03.2025	Canidae
**PV738449**	H5N1	2025AI02248	Germany-SN	17.03.2025	Canidae
**PV738450**	H5N1	2025AI02248	Germany-SN	17.03.2025	Canidae
**PV738451**	H5N1	2025AI02248	Germany-SN	17.03.2025	Canidae
**PV738452**	H5N1	2025AI02248	Germany-SN	17.03.2025	Canidae
**PV738453**	H5N1	2025AI02248	Germany-SN	17.03.2025	Canidae
**PV738454**	H5N1	2025AI02248	Germany-SN	17.03.2025	Canidae
**PV738455**	H5N1	2025AI02363	Germany-NI	04.03.2025	Anatidae
**PV738456**	H5N1	2025AI02363	Germany-NI	04.03.2025	Anatidae
**PV738457**	H5N1	2025AI02363	Germany-NI	04.03.2025	Anatidae
**PV738458**	H5N1	2025AI02363	Germany-NI	04.03.2025	Anatidae
**PV738459**	H5N1	2025AI02363	Germany-NI	04.03.2025	Anatidae
**PV738460**	H5N1	2025AI02363	Germany-NI	04.03.2025	Anatidae
**PV738461**	H5N1	2025AI02363	Germany-NI	04.03.2025	Anatidae
**PV738462**	H5N1	2025AI02363	Germany-NI	04.03.2025	Anatidae
**PV738463**	H5N1	2025AI02206	Germany-NI	17.03.2025	raptor
**PV738464**	H5N1	2025AI02206	Germany-NI	17.03.2025	raptor

**PV738465**	H5N1	2025AI02206	Germany-NI	17.03.2025	raptor
**PV738466**	H5N1	2025AI02206	Germany-NI	17.03.2025	raptor
**PV738467**	H5N1	2025AI02206	Germany-NI	17.03.2025	raptor
**PV738468**	H5N1	2025AI02206	Germany-NI	17.03.2025	raptor
**PV738469**	H5N1	2025AI02206	Germany-NI	17.03.2025	raptor
**PV738470**	H5N1	2025AI02206	Germany-NI	17.03.2025	raptor
**PV738471**	H5N1	2025AI02372	Germany-MV	26.03.2025	Vulpes vulpes
**PV738472**	H5N1	2025AI02372	Germany-MV	26.03.2025	Vulpes vulpes
**PV738473**	H5N1	2025AI02372	Germany-MV	26.03.2025	Vulpes vulpes
**PV738474**	H5N1	2025AI02372	Germany-MV	26.03.2025	Vulpes vulpes
**PV738475**	H5N1	2025AI02372	Germany-MV	26.03.2025	Vulpes vulpes
**PV738476**	H5N1	2025AI02372	Germany-MV	26.03.2025	Vulpes vulpes
**PV738477**	H5N1	2025AI02372	Germany-MV	26.03.2025	Vulpes vulpes
**PV738478**	H5N1	2025AI02372	Germany-MV	26.03.2025	Vulpes vulpes
**PV738479**	H5N1	2025AI02097	Germany-TH	09.03.2025	Anatidae
**PV738480**	H5N1	2025AI02097	Germany-TH	09.03.2025	Anatidae
**PV738481**	H5N1	2025AI02097	Germany-TH	09.03.2025	Anatidae
**PV738482**	H5N1	2025AI02097	Germany-TH	09.03.2025	Anatidae
**PV738483**	H5N1	2025AI02097	Germany-TH	09.03.2025	Anatidae
**PV738484**	H5N1	2025AI02097	Germany-TH	09.03.2025	Anatidae
**PV738485**	H5N1	2025AI02097	Germany-TH	09.03.2025	Anatidae
**PV738486**	H5N1	2025AI02097	Germany-TH	09.03.2025	Anatidae
**PV738526**	H5N1	2025AI02099	Germany-BY	12.03.2025	Meleagris gallopavo
**PV738527**	H5N1	2025AI02099	Germany-BY	12.03.2025	Meleagris gallopavo
**PV738528**	H5N1	2025AI02099	Germany-BY	12.03.2025	Meleagris gallopavo
**PV738529**	H5N1	2025AI02099	Germany-BY	12.03.2025	Meleagris gallopavo
**PV738530**	H5N1	2025AI02099	Germany-BY	12.03.2025	Meleagris gallopavo
**PV738531**	H5N1	2025AI02099	Germany-BY	12.03.2025	Meleagris gallopavo
**PV738532**	H5N1	2025AI02099	Germany-BY	12.03.2025	Meleagris gallopavo
**PV738533**	H5N1	2025AI02099	Germany-BY	12.03.2025	Meleagris gallopavo
**PV738534**	H5N1	2025AI01378	Germany-BB	17.02.2025	Gallus gallus
**PV738535**	H5N1	2025AI01378	Germany-BB	17.02.2025	Gallus gallus
**PV738536**	H5N1	2025AI01378	Germany-BB	17.02.2025	Gallus gallus
**PV738537**	H5N1	2025AI01378	Germany-BB	17.02.2025	Gallus gallus
**PV738538**	H5N1	2025AI01378	Germany-BB	17.02.2025	Gallus gallus
**PV738539**	H5N1	2025AI01378	Germany-BB	17.02.2025	Gallus gallus
**PV738540**	H5N1	2025AI01378	Germany-BB	17.02.2025	Gallus gallus
**PV738541**	H5N1	2025AI01378	Germany-BB	17.02.2025	Gallus gallus
**PV738542**	H5N1	2025AI01384	Germany-NI	22.02.2025	Gallus gallus
**PV738543**	H5N1	2025AI01384	Germany-NI	22.02.2025	Gallus gallus
**PV738544**	H5N1	2025AI01384	Germany-NI	22.02.2025	Gallus gallus
**PV738545**	H5N1	2025AI01384	Germany-NI	22.02.2025	Gallus gallus
**PV738546**	H5N1	2025AI01384	Germany-NI	22.02.2025	Gallus gallus
**PV738547**	H5N1	2025AI01384	Germany-NI	22.02.2025	Gallus gallus
**PV738548**	H5N1	2025AI01384	Germany-NI	22.02.2025	Gallus gallus

**PV738549**	H5N1	2025AI01384	Germany-NI	22.02.2025	Gallus gallus
**PV738550**	H5N1	2025AI02097	Germany-TH	09.03.2025	Anatidae
**PV738551**	H5N1	2025AI02097	Germany-TH	09.03.2025	Anatidae
**PV738552**	H5N1	2025AI02097	Germany-TH	09.03.2025	Anatidae
**PV738553**	H5N1	2025AI02097	Germany-TH	09.03.2025	Anatidae
**PV738554**	H5N1	2025AI02097	Germany-TH	09.03.2025	Anatidae
**PV738555**	H5N1	2025AI02097	Germany-TH	09.03.2025	Anatidae
**PV738556**	H5N1	2025AI02097	Germany-TH	09.03.2025	Anatidae
**PV738557**	H5N1	2025AI02097	Germany-TH	09.03.2025	Anatidae
**PV242319**	H5N1	2025AI00862	Germany-NI	28.01.2025	Gallus gallus
**PV242320**	H5N1	2025AI00862	Germany-NI	28.01.2025	Gallus gallus
**PV242321**	H5N1	2025AI00862	Germany-NI	28.01.2025	Gallus gallus
**PV242322**	H5N1	2025AI00862	Germany-NI	28.01.2025	Gallus gallus
**PV242323**	H5N1	2025AI00862	Germany-NI	28.01.2025	Gallus gallus
**PV242324**	H5N1	2025AI00862	Germany-NI	28.01.2025	Gallus gallus
**PV242325**	H5N1	2025AI00862	Germany-NI	28.01.2025	Gallus gallus
**PV242326**	H5N1	2025AI00862	Germany-NI	28.01.2025	Gallus gallus
**PV242327**	H5N1	2025AI00855	Germany-NI	17.01.2025	Branta leucopsis
**PV242328**	H5N1	2025AI00855	Germany-NI	17.01.2025	Branta leucopsis
**PV242329**	H5N1	2025AI00855	Germany-NI	17.01.2025	Branta leucopsis
**PV242330**	H5N1	2025AI00855	Germany-NI	17.01.2025	Branta leucopsis
**PV242331**	H5N1	2025AI00855	Germany-NI	17.01.2025	Branta leucopsis
**PV242332**	H5N1	2025AI00855	Germany-NI	17.01.2025	Branta leucopsis
**PV242333**	H5N1	2025AI00855	Germany-NI	17.01.2025	Branta leucopsis
**PV242334**	H5N1	2025AI00855	Germany-NI	17.01.2025	Branta leucopsis
**PV242335**	H5N1	2025AI00861	Germany-ST	24.01.2025	Anatidae
**PV242336**	H5N1	2025AI00861	Germany-ST	24.01.2025	Anatidae
**PV242337**	H5N1	2025AI00861	Germany-ST	24.01.2025	Anatidae
**PV242338**	H5N1	2025AI00861	Germany-ST	24.01.2025	Anatidae
**PV242339**	H5N1	2025AI00861	Germany-ST	24.01.2025	Anatidae
**PV242340**	H5N1	2025AI00861	Germany-ST	24.01.2025	Anatidae
**PV242341**	H5N1	2025AI00861	Germany-ST	24.01.2025	Anatidae
**PV242342**	H5N1	2025AI00861	Germany-ST	24.01.2025	Anatidae
**PV242343**	H5N1	2025AI01244	Germany-NI	06.02.2025	Branta leucopsis
**PV242344**	H5N1	2025AI01244	Germany-NI	06.02.2025	Branta leucopsis
**PV242345**	H5N1	2025AI01244	Germany-NI	06.02.2025	Branta leucopsis
**PV242346**	H5N1	2025AI01244	Germany-NI	06.02.2025	Branta leucopsis
**PV242347**	H5N1	2025AI01244	Germany-NI	06.02.2025	Branta leucopsis
**PV242348**	H5N1	2025AI01244	Germany-NI	06.02.2025	Branta leucopsis
**PV242349**	H5N1	2025AI01244	Germany-NI	06.02.2025	Branta leucopsis
**PV242350**	H5N1	2025AI01244	Germany-NI	06.02.2025	Branta leucopsis
**PV242351**	H5N1	2025AI01255	Germany-BY	04.02.2025	Anser sp.
**PV242352**	H5N1	2025AI01255	Germany-BY	04.02.2025	Anser sp.
**PV242353**	H5N1	2025AI01255	Germany-BY	04.02.2025	Anser sp.
**PV242354**	H5N1	2025AI01255	Germany-BY	04.02.2025	Anser sp.

**PV242355**	H5N1	2025AI01255	Germany-BY	04.02.2025	Anser sp.
**PV242356**	H5N1	2025AI01255	Germany-BY	04.02.2025	Anser sp.
**PV242357**	H5N1	2025AI01255	Germany-BY	04.02.2025	Anser sp.
**PV242358**	H5N1	2025AI01255	Germany-BY	04.02.2025	Anser sp.
**PV242359**	H5N1	2025AI01260	Germany-MV	07.02.2025	Gallus gallus
**PV242360**	H5N1	2025AI01260	Germany-MV	07.02.2025	Gallus gallus
**PV242361**	H5N1	2025AI01260	Germany-MV	07.02.2025	Gallus gallus
**PV242362**	H5N1	2025AI01260	Germany-MV	07.02.2025	Gallus gallus
**PV242363**	H5N1	2025AI01260	Germany-MV	07.02.2025	Gallus gallus
**PV242364**	H5N1	2025AI01260	Germany-MV	07.02.2025	Gallus gallus
**PV242365**	H5N1	2025AI01260	Germany-MV	07.02.2025	Gallus gallus
**PV242366**	H5N1	2025AI01260	Germany-MV	07.02.2025	Gallus gallus
**PV242367**	H5N1	2025AI01274	Germany-ST	07.02.2025	Ardea cinerea
**PV242368**	H5N1	2025AI01274	Germany-ST	07.02.2025	Ardea cinerea
**PV242369**	H5N1	2025AI01274	Germany-ST	07.02.2025	Ardea cinerea
**PV242370**	H5N1	2025AI01274	Germany-ST	07.02.2025	Ardea cinerea
**PV242371**	H5N1	2025AI01274	Germany-ST	07.02.2025	Ardea cinerea
**PV242372**	H5N1	2025AI01274	Germany-ST	07.02.2025	Ardea cinerea
**PV242373**	H5N1	2025AI01274	Germany-ST	07.02.2025	Ardea cinerea
**PV242374**	H5N1	2025AI01274	Germany-ST	07.02.2025	Ardea cinerea
**PV242375**	H5N1	2025AI01283	Germany-BY	12.02.2025	Branta canadensis
**PV242376**	H5N1	2025AI01283	Germany-BY	12.02.2025	Branta canadensis
**PV242377**	H5N1	2025AI01283	Germany-BY	12.02.2025	Branta canadensis
**PV242378**	H5N1	2025AI01283	Germany-BY	12.02.2025	Branta canadensis
**PV242379**	H5N1	2025AI01283	Germany-BY	12.02.2025	Branta canadensis
**PV242380**	H5N1	2025AI01283	Germany-BY	12.02.2025	Branta canadensis
**PV242381**	H5N1	2025AI01283	Germany-BY	12.02.2025	Branta canadensis
**PV242382**	H5N1	2025AI01283	Germany-BY	12.02.2025	Branta canadensis
**PV242383**	H5N1	2025AI00851	Germany-NW	18.01.2025	Anatidae
**PV242384**	H5N1	2025AI00851	Germany-NW	18.01.2025	Anatidae
**PV242385**	H5N1	2025AI00851	Germany-NW	18.01.2025	Anatidae
**PV242386**	H5N1	2025AI00851	Germany-NW	18.01.2025	Anatidae
**PV242387**	H5N1	2025AI00851	Germany-NW	18.01.2025	Anatidae
**PV242388**	H5N1	2025AI00851	Germany-NW	18.01.2025	Anatidae
**PV242389**	H5N1	2025AI00851	Germany-NW	18.01.2025	Anatidae
**PV242390**	H5N1	2025AI00851	Germany-NW	18.01.2025	Anatidae
**PV187756**	H5N1	2024AI05018	Germany-MV	25.11.2024	Anas platyrhynchos
**PV187757**	H5N1	2024AI05018	Germany-MV	25.11.2024	Anas platyrhynchos
**PV187758**	H5N1	2024AI05018	Germany-MV	25.11.2024	Anas platyrhynchos
**PV187759**	H5N1	2024AI05018	Germany-MV	25.11.2024	Anas platyrhynchos
**PV187760**	H5N1	2024AI05018	Germany-MV	25.11.2024	Anas platyrhynchos
**PV187761**	H5N1	2024AI05018	Germany-MV	25.11.2024	Anas platyrhynchos
**PV187762**	H5N1	2024AI05018	Germany-MV	25.11.2024	Anas platyrhynchos
**PV187763**	H5N1	2024AI05018	Germany-MV	25.11.2024	Anas platyrhynchos
**PV187764**	H5N1	2024AI05027	Germany-SH	18.11.2024	Mareca penelope

**PV187765**	H5N1	2024AI05027	Germany-SH	18.11.2024	Mareca penelope
**PV187766**	H5N1	2024AI05027	Germany-SH	18.11.2024	Mareca penelope
**PV187767**	H5N1	2024AI05027	Germany-SH	18.11.2024	Mareca penelope
**PV187768**	H5N1	2024AI05027	Germany-SH	18.11.2024	Mareca penelope
**PV187769**	H5N1	2024AI05027	Germany-SH	18.11.2024	Mareca penelope
**PV187770**	H5N1	2024AI05027	Germany-SH	18.11.2024	Mareca penelope
**PV187771**	H5N1	2024AI05027	Germany-SH	18.11.2024	Mareca penelope
**PV187772**	H5N1	2024AI05042	Germany-NI	22.11.2024	Branta leucopsis
**PV187773**	H5N1	2024AI05042	Germany-NI	22.11.2024	Branta leucopsis
**PV187774**	H5N1	2024AI05042	Germany-NI	22.11.2024	Branta leucopsis
**PV187775**	H5N1	2024AI05042	Germany-NI	22.11.2024	Branta leucopsis
**PV187776**	H5N1	2024AI05042	Germany-NI	22.11.2024	Branta leucopsis
**PV187777**	H5N1	2024AI05042	Germany-NI	22.11.2024	Branta leucopsis
**PV187778**	H5N1	2024AI05042	Germany-NI	22.11.2024	Branta leucopsis
**PV187779**	H5N1	2024AI05042	Germany-NI	22.11.2024	Branta leucopsis
**PV187780**	H5N1	2024AI05044	Germany-HH	28.11.2024	Laridae
**PV187781**	H5N1	2024AI05044	Germany-HH	28.11.2024	Laridae
**PV187782**	H5N1	2024AI05044	Germany-HH	28.11.2024	Laridae
**PV187783**	H5N1	2024AI05044	Germany-HH	28.11.2024	Laridae
**PV187784**	H5N1	2024AI05044	Germany-HH	28.11.2024	Laridae
**PV187785**	H5N1	2024AI05044	Germany-HH	28.11.2024	Laridae
**PV187786**	H5N1	2024AI05044	Germany-HH	28.11.2024	Laridae
**PV187787**	H5N1	2024AI05044	Germany-HH	28.11.2024	Laridae
**PV187788**	H5N1	2024AI05060	Germany-SH	26.11.2024	Larus marinus
**PV187789**	H5N1	2024AI05060	Germany-SH	26.11.2024	Larus marinus
**PV187790**	H5N1	2024AI05060	Germany-SH	26.11.2024	Larus marinus
**PV187791**	H5N1	2024AI05060	Germany-SH	26.11.2024	Larus marinus
**PV187792**	H5N1	2024AI05060	Germany-SH	26.11.2024	Larus marinus
**PV187793**	H5N1	2024AI05060	Germany-SH	26.11.2024	Larus marinus
**PV187794**	H5N1	2024AI05060	Germany-SH	26.11.2024	Larus marinus
**PV187795**	H5N1	2024AI05060	Germany-SH	26.11.2024	Larus marinus
**PV187796**	H5N1	2024AI05071	Germany-MV	06.12.2024	Meleagris gallopavo
**PV187797**	H5N1	2024AI05071	Germany-MV	06.12.2024	Meleagris gallopavo
**PV187798**	H5N1	2024AI05071	Germany-MV	06.12.2024	Meleagris gallopavo
**PV187799**	H5N1	2024AI05071	Germany-MV	06.12.2024	Meleagris gallopavo
**PV187800**	H5N1	2024AI05071	Germany-MV	06.12.2024	Meleagris gallopavo
**PV187801**	H5N1	2024AI05071	Germany-MV	06.12.2024	Meleagris gallopavo
**PV187802**	H5N1	2024AI05071	Germany-MV	06.12.2024	Meleagris gallopavo
**PV187803**	H5N1	2024AI05071	Germany-MV	06.12.2024	Meleagris gallopavo
**PV187804**	H5N1	2024AI05112	Germany-NI	02.12.2024	Anser brachyrhynchus
**PV187805**	H5N1	2024AI05112	Germany-NI	02.12.2024	Anser brachyrhynchus
**PV187806**	H5N1	2024AI05112	Germany-NI	02.12.2024	Anser brachyrhynchus
**PV187807**	H5N1	2024AI05112	Germany-NI	02.12.2024	Anser brachyrhynchus
**PV187808**	H5N1	2024AI05112	Germany-NI	02.12.2024	Anser brachyrhynchus
**PV187809**	H5N1	2024AI05112	Germany-NI	02.12.2024	Anser brachyrhynchus

**PV187810**	H5N1	2024AI05112	Germany-NI	02.12.2024	Anser brachyrhynchus
**PV187811**	H5N1	2024AI05112	Germany-NI	02.12.2024	Anser brachyrhynchus
**PV187812**	H5N1	2024AI05114	Germany-BW	09.12.2024	Anatidae
**PV187813**	H5N1	2024AI05114	Germany-BW	09.12.2024	Anatidae
**PV187814**	H5N1	2024AI05114	Germany-BW	09.12.2024	Anatidae
**PV187815**	H5N1	2024AI05114	Germany-BW	09.12.2024	Anatidae
**PV187816**	H5N1	2024AI05138	Germany-BW	11.12.2024	Ciconia ciconia
**PV187817**	H5N1	2024AI05138	Germany-BW	11.12.2024	Ciconia ciconia
**PV187818**	H5N1	2024AI05138	Germany-BW	11.12.2024	Ciconia ciconia
**PV187819**	H5N1	2024AI05138	Germany-BW	11.12.2024	Ciconia ciconia
**PV187820**	H5N1	2024AI05138	Germany-BW	11.12.2024	Ciconia ciconia
**PV187821**	H5N1	2024AI05138	Germany-BW	11.12.2024	Ciconia ciconia
**PV187822**	H5N1	2024AI05138	Germany-BW	11.12.2024	Ciconia ciconia
**PV187823**	H5N1	2024AI05138	Germany-BW	11.12.2024	Ciconia ciconia
**PV187824**	H5N1	2024AI05146	Germany-SH	02.12.2024	Branta leucopsis
**PV187825**	H5N1	2024AI05146	Germany-SH	02.12.2024	Branta leucopsis
**PV187826**	H5N1	2024AI05146	Germany-SH	02.12.2024	Branta leucopsis
**PV187827**	H5N1	2024AI05146	Germany-SH	02.12.2024	Branta leucopsis
**PV187828**	H5N1	2024AI05146	Germany-SH	02.12.2024	Branta leucopsis
**PV187829**	H5N1	2024AI05146	Germany-SH	02.12.2024	Branta leucopsis
**PV187830**	H5N1	2024AI05146	Germany-SH	02.12.2024	Branta leucopsis
**PV187831**	H5N1	2024AI05146	Germany-SH	02.12.2024	Branta leucopsis
**PV187832**	H5N1	2024AI05148	Germany-BW	16.12.2024	Gallus gallus
**PV187833**	H5N1	2024AI05148	Germany-BW	16.12.2024	Gallus gallus
**PV187834**	H5N1	2024AI05148	Germany-BW	16.12.2024	Gallus gallus
**PV187835**	H5N1	2024AI05148	Germany-BW	16.12.2024	Gallus gallus
**PV187836**	H5N1	2024AI05148	Germany-BW	16.12.2024	Gallus gallus
**PV187837**	H5N1	2024AI05148	Germany-BW	16.12.2024	Gallus gallus
**PV187838**	H5N1	2024AI05148	Germany-BW	16.12.2024	Gallus gallus
**PV187839**	H5N1	2024AI05148	Germany-BW	16.12.2024	Gallus gallus
**PV187840**	H5N1	2024AI05149	Germany-MV	22.12.2024	Gallus gallus
**PV187841**	H5N1	2024AI05149	Germany-MV	22.12.2024	Gallus gallus
**PV187842**	H5N1	2024AI05149	Germany-MV	22.12.2024	Gallus gallus
**PV187843**	H5N1	2024AI05149	Germany-MV	22.12.2024	Gallus gallus
**PV187844**	H5N1	2024AI05149	Germany-MV	22.12.2024	Gallus gallus
**PV187845**	H5N1	2024AI05149	Germany-MV	22.12.2024	Gallus gallus
**PV187846**	H5N1	2024AI05149	Germany-MV	22.12.2024	Gallus gallus
**PV187847**	H5N1	2024AI05149	Germany-MV	22.12.2024	Gallus gallus
**PV187848**	H5N1	2024AI05156	Germany-MV	27.12.2024	Cereopsis novaehollandiae
**PV187849**	H5N1	2024AI05156	Germany-MV	27.12.2024	Cereopsis novaehollandiae
**PV187850**	H5N1	2024AI05156	Germany-MV	27.12.2024	Cereopsis novaehollandiae
**PV187851**	H5N1	2024AI05156	Germany-MV	27.12.2024	Cereopsis novaehollandiae
**PV187852**	H5N1	2024AI05156	Germany-MV	27.12.2024	Cereopsis novaehollandiae
**PV187853**	H5N1	2024AI05156	Germany-MV	27.12.2024	Cereopsis novaehollandiae
**PV187854**	H5N1	2024AI05156	Germany-MV	27.12.2024	Cereopsis novaehollandiae

**PV187855**	H5N1	2024AI05156	Germany-MV	27.12.2024	Cereopsis novaehollandiae
**PV187856**	H5N1	2024AI05159	Germany-BY	27.12.2024	Aix galericulata
**PV187857**	H5N1	2024AI05159	Germany-BY	27.12.2024	Aix galericulata
**PV187858**	H5N1	2024AI05159	Germany-BY	27.12.2024	Aix galericulata
**PV187859**	H5N1	2024AI05159	Germany-BY	27.12.2024	Aix galericulata
**PV187860**	H5N1	2024AI05159	Germany-BY	27.12.2024	Aix galericulata
**PV187861**	H5N1	2024AI05159	Germany-BY	27.12.2024	Aix galericulata
**PV187862**	H5N1	2024AI05159	Germany-BY	27.12.2024	Aix galericulata
**PV187863**	H5N1	2024AI05159	Germany-BY	27.12.2024	Aix galericulata
**PV187864**	H5N1	2025AI00004	Germany-MV	31.12.2024	Meleagris gallopavo
**PV187865**	H5N1	2025AI00004	Germany-MV	31.12.2024	Meleagris gallopavo
**PV187866**	H5N1	2025AI00004	Germany-MV	31.12.2024	Meleagris gallopavo
**PV187867**	H5N1	2025AI00004	Germany-MV	31.12.2024	Meleagris gallopavo
**PV187868**	H5N1	2025AI00004	Germany-MV	31.12.2024	Meleagris gallopavo
**PV187869**	H5N1	2025AI00004	Germany-MV	31.12.2024	Meleagris gallopavo
**PV187870**	H5N1	2025AI00004	Germany-MV	31.12.2024	Meleagris gallopavo
**PV187871**	H5N1	2025AI00004	Germany-MV	31.12.2024	Meleagris gallopavo
**PV187872**	H5N5	2025AI00015	Germany-SH	10.12.2024	Larus canus
**PV187873**	H5N5	2025AI00015	Germany-SH	10.12.2024	Larus canus
**PV187874**	H5N5	2025AI00015	Germany-SH	10.12.2024	Larus canus
**PV187875**	H5N5	2025AI00015	Germany-SH	10.12.2024	Larus canus
**PV187876**	H5N5	2025AI00015	Germany-SH	10.12.2024	Larus canus
**PV187877**	H5N5	2025AI00015	Germany-SH	10.12.2024	Larus canus
**PV187878**	H5N5	2025AI00015	Germany-SH	10.12.2024	Larus canus
**PV187879**	H5N5	2025AI00015	Germany-SH	10.12.2024	Larus canus
**PV187880**	H5N1	2025AI00022	Germany-SH	12.12.2024	Cygnus olor
**PV187881**	H5N1	2025AI00022	Germany-SH	12.12.2024	Cygnus olor
**PV187882**	H5N1	2025AI00022	Germany-SH	12.12.2024	Cygnus olor
**PV187883**	H5N1	2025AI00022	Germany-SH	12.12.2024	Cygnus olor
**PV187884**	H5N1	2025AI00022	Germany-SH	12.12.2024	Cygnus olor
**PV187885**	H5N1	2025AI00022	Germany-SH	12.12.2024	Cygnus olor
**PV187886**	H5N1	2025AI00022	Germany-SH	12.12.2024	Cygnus olor
**PV187887**	H5N1	2025AI00022	Germany-SH	12.12.2024	Cygnus olor
**PV187888**	H5N1	2025AI00031	Germany-SH	06.01.2025	Gallus gallus
**PV187889**	H5N1	2025AI00031	Germany-SH	06.01.2025	Gallus gallus
**PV187890**	H5N1	2025AI00031	Germany-SH	06.01.2025	Gallus gallus
**PV187891**	H5N1	2025AI00031	Germany-SH	06.01.2025	Gallus gallus
**PV187892**	H5N1	2025AI00031	Germany-SH	06.01.2025	Gallus gallus
**PV187893**	H5N1	2025AI00031	Germany-SH	06.01.2025	Gallus gallus
**PV187894**	H5N1	2025AI00031	Germany-SH	06.01.2025	Gallus gallus
**PV187895**	H5N1	2025AI00031	Germany-SH	06.01.2025	Gallus gallus
**PV187896**	H5N1	2025AI00038	Germany-SH	02.01.2025	Mareca penelope
**PV187897**	H5N1	2025AI00038	Germany-SH	02.01.2025	Mareca penelope
**PV187898**	H5N1	2025AI00038	Germany-SH	02.01.2025	Mareca penelope
**PV187899**	H5N1	2025AI00038	Germany-SH	02.01.2025	Mareca penelope

**PV187900**	H5N1	2025AI00038	Germany-SH	02.01.2025	Mareca penelope
**PV187901**	H5N1	2025AI00038	Germany-SH	02.01.2025	Mareca penelope
**PV187902**	H5N1	2025AI00038	Germany-SH	02.01.2025	Mareca penelope
**PV187903**	H5N1	2025AI00038	Germany-SH	02.01.2025	Mareca penelope
**PV187904**	H5N1	2025AI00175	Germany-NI	11.01.2025	Meleagris gallopavo
**PV187905**	H5N1	2025AI00175	Germany-NI	11.01.2025	Meleagris gallopavo
**PV187906**	H5N1	2025AI00175	Germany-NI	11.01.2025	Meleagris gallopavo
**PV187907**	H5N1	2025AI00175	Germany-NI	11.01.2025	Meleagris gallopavo
**PV187908**	H5N1	2025AI00175	Germany-NI	11.01.2025	Meleagris gallopavo
**PV187909**	H5N1	2025AI00175	Germany-NI	11.01.2025	Meleagris gallopavo
**PV187910**	H5N1	2025AI00175	Germany-NI	11.01.2025	Meleagris gallopavo
**PV187911**	H5N1	2025AI00175	Germany-NI	11.01.2025	Meleagris gallopavo
**PV187912**	H5N1	2024AI05053	Germany-NI	03.12.2024	Branta leucopsis
**PV187913**	H5N1	2024AI05053	Germany-NI	03.12.2024	Branta leucopsis
**PV187914**	H5N1	2024AI05053	Germany-NI	03.12.2024	Branta leucopsis
**PV187915**	H5N1	2024AI05053	Germany-NI	03.12.2024	Branta leucopsis
**PV187916**	H5N1	2024AI05053	Germany-NI	03.12.2024	Branta leucopsis
**PV187917**	H5N1	2024AI05053	Germany-NI	03.12.2024	Branta leucopsis
**PV187918**	H5N1	2024AI05053	Germany-NI	03.12.2024	Branta leucopsis
**PV187919**	H5N1	2024AI05053	Germany-NI	03.12.2024	Branta leucopsis
**PV187920**	H5N1	2024AI05048	Germany-MV	02.12.2024	Mareca strepera
**PV187921**	H5N1	2024AI05048	Germany-MV	02.12.2024	Mareca strepera
**PV187922**	H5N1	2024AI05048	Germany-MV	02.12.2024	Mareca strepera
**PV187923**	H5N1	2024AI05048	Germany-MV	02.12.2024	Mareca strepera
**PV187924**	H5N1	2024AI05048	Germany-MV	02.12.2024	Mareca strepera
**PV187925**	H5N1	2024AI05048	Germany-MV	02.12.2024	Mareca strepera
**PV187926**	H5N1	2024AI05048	Germany-MV	02.12.2024	Mareca strepera
**PV187927**	H5N1	2024AI05048	Germany-MV	02.12.2024	Mareca strepera
**PV187928**	H5N1	2024AI05041	Germany-BY	25.11.2024	Ardea alba
**PV187929**	H5N1	2024AI05041	Germany-BY	25.11.2024	Ardea alba
**PV187930**	H5N1	2024AI05041	Germany-BY	25.11.2024	Ardea alba
**PV187931**	H5N1	2024AI05041	Germany-BY	25.11.2024	Ardea alba
**PV187932**	H5N1	2024AI05041	Germany-BY	25.11.2024	Ardea alba
**PV187933**	H5N1	2024AI05041	Germany-BY	25.11.2024	Ardea alba
**PV187934**	H5N1	2024AI05041	Germany-BY	25.11.2024	Ardea alba
**PV187935**	H5N1	2024AI05041	Germany-BY	25.11.2024	Ardea alba
**PV187936**	H5N1	2024AI05162	Germany-BY	27.12.2024	Pavo cristatus
**PV187937**	H5N1	2024AI05162	Germany-BY	27.12.2024	Pavo cristatus
**PV187938**	H5N1	2024AI05162	Germany-BY	27.12.2024	Pavo cristatus
**PV187939**	H5N1	2024AI05162	Germany-BY	27.12.2024	Pavo cristatus
**PV187940**	H5N1	2024AI05162	Germany-BY	27.12.2024	Pavo cristatus
**PV187941**	H5N1	2024AI05162	Germany-BY	27.12.2024	Pavo cristatus
**PV187942**	H5N1	2024AI05162	Germany-BY	27.12.2024	Pavo cristatus
**PV187943**	H5N1	2024AI05162	Germany-BY	27.12.2024	Pavo cristatus
**PV187944**	H5N1	2024AI05139	Germany-BB	06.12.2024	Falco peregrinus

**PV187945**	H5N1	2024AI05139	Germany-BB	06.12.2024	Falco peregrinus
**PV187946**	H5N1	2024AI05139	Germany-BB	06.12.2024	Falco peregrinus
**PV187947**	H5N1	2024AI05139	Germany-BB	06.12.2024	Falco peregrinus
**PV187948**	H5N1	2024AI05139	Germany-BB	06.12.2024	Falco peregrinus
**PV187949**	H5N1	2024AI05139	Germany-BB	06.12.2024	Falco peregrinus
**PV187950**	H5N1	2024AI05139	Germany-BB	06.12.2024	Falco peregrinus
**PV187951**	H5N1	2024AI05139	Germany-BB	06.12.2024	Falco peregrinus
**PV187952**	H5N1	2025AI00446	Germany-BY	14.01.2025	Ciconia ciconia
**PV187953**	H5N1	2025AI00446	Germany-BY	14.01.2025	Ciconia ciconia
**PV187954**	H5N1	2025AI00446	Germany-BY	14.01.2025	Ciconia ciconia
**PV187955**	H5N1	2025AI00446	Germany-BY	14.01.2025	Ciconia ciconia
**PV187956**	H5N1	2025AI00446	Germany-BY	14.01.2025	Ciconia ciconia
**PV187957**	H5N1	2025AI00446	Germany-BY	14.01.2025	Ciconia ciconia
**PV187958**	H5N1	2025AI00446	Germany-BY	14.01.2025	Ciconia ciconia
**PV187959**	H5N1	2025AI00446	Germany-BY	14.01.2025	Ciconia ciconia
**PV187960**	H5N1	2024AI05017	Germany-MV	25.11.2024	Anas platyrhynchos
**PV187961**	H5N1	2024AI05017	Germany-MV	25.11.2024	Anas platyrhynchos
**PV187962**	H5N1	2024AI05017	Germany-MV	25.11.2024	Anas platyrhynchos
**PV187963**	H5N1	2024AI05017	Germany-MV	25.11.2024	Anas platyrhynchos
**PV187964**	H5N1	2024AI05017	Germany-MV	25.11.2024	Anas platyrhynchos
**PV187965**	H5N1	2024AI05017	Germany-MV	25.11.2024	Anas platyrhynchos
**PV187966**	H5N1	2024AI05017	Germany-MV	25.11.2024	Anas platyrhynchos
**PV187967**	H5N1	2024AI05017	Germany-MV	25.11.2024	Anas platyrhynchos
**PV187968**	H5N1	2024AI05172	Germany-RP	28.12.2024	Anatidae
**PV187969**	H5N1	2024AI05172	Germany-RP	28.12.2024	Anatidae
**PV187970**	H5N1	2024AI05172	Germany-RP	28.12.2024	Anatidae
**PV187971**	H5N1	2024AI05172	Germany-RP	28.12.2024	Anatidae
**PV187972**	H5N1	2024AI05172	Germany-RP	28.12.2024	Anatidae
**PV187973**	H5N1	2024AI05172	Germany-RP	28.12.2024	Anatidae
**PV187974**	H5N1	2024AI05172	Germany-RP	28.12.2024	Anatidae
**PV187975**	H5N1	2024AI05172	Germany-RP	28.12.2024	Anatidae
**PV187976**	H5N1	2025AI00222	Germany-BW	12.01.2025	Meleagris gallopavo
**PV187977**	H5N1	2025AI00222	Germany-BW	12.01.2025	Meleagris gallopavo
**PV187978**	H5N1	2025AI00222	Germany-BW	12.01.2025	Meleagris gallopavo
**PV187979**	H5N1	2025AI00222	Germany-BW	12.01.2025	Meleagris gallopavo
**PV187980**	H5N1	2025AI00222	Germany-BW	12.01.2025	Meleagris gallopavo
**PV187981**	H5N1	2025AI00222	Germany-BW	12.01.2025	Meleagris gallopavo
**PV187982**	H5N1	2025AI00222	Germany-BW	12.01.2025	Meleagris gallopavo
**PV187983**	H5N1	2025AI00222	Germany-BW	12.01.2025	Meleagris gallopavo
**PV187984**	H5N1	2024AI05169	Germany-BY	23.12.2024	Meleagris gallopavo
**PV187985**	H5N1	2024AI05169	Germany-BY	23.12.2024	Meleagris gallopavo
**PV187986**	H5N1	2024AI05169	Germany-BY	23.12.2024	Meleagris gallopavo
**PV187987**	H5N1	2024AI05169	Germany-BY	23.12.2024	Meleagris gallopavo
**PV187988**	H5N1	2024AI05169	Germany-BY	23.12.2024	Meleagris gallopavo
**PV187989**	H5N1	2024AI05169	Germany-BY	23.12.2024	Meleagris gallopavo

**PV187990**	H5N1	2024AI05169	Germany-BY	23.12.2024	Meleagris gallopavo
**PV187991**	H5N1	2024AI05169	Germany-BY	23.12.2024	Meleagris gallopavo
**PV187992**	H5N1	2024AI05038	Germany-NI	28.11.2024	Meleagris gallopavo
**PV187993**	H5N1	2024AI05038	Germany-NI	28.11.2024	Meleagris gallopavo
**PV187994**	H5N1	2024AI05038	Germany-NI	28.11.2024	Meleagris gallopavo
**PV187995**	H5N1	2024AI05038	Germany-NI	28.11.2024	Meleagris gallopavo
**PV187996**	H5N1	2024AI05038	Germany-NI	28.11.2024	Meleagris gallopavo
**PV187997**	H5N1	2024AI05038	Germany-NI	28.11.2024	Meleagris gallopavo
**PV187998**	H5N1	2024AI05038	Germany-NI	28.11.2024	Meleagris gallopavo
**PV187999**	H5N1	2024AI05038	Germany-NI	28.11.2024	Meleagris gallopavo
**PV188000**	H5N1	2024AI05064	Germany-NI	04.12.2024	Meleagris gallopavo
**PV188001**	H5N1	2024AI05064	Germany-NI	04.12.2024	Meleagris gallopavo
**PV188002**	H5N1	2024AI05064	Germany-NI	04.12.2024	Meleagris gallopavo
**PV188003**	H5N1	2024AI05064	Germany-NI	04.12.2024	Meleagris gallopavo
**PV188004**	H5N1	2024AI05064	Germany-NI	04.12.2024	Meleagris gallopavo
**PV188005**	H5N1	2024AI05064	Germany-NI	04.12.2024	Meleagris gallopavo
**PV188006**	H5N1	2024AI05064	Germany-NI	04.12.2024	Meleagris gallopavo
**PV188007**	H5N1	2024AI05064	Germany-NI	04.12.2024	Meleagris gallopavo
**PV188008**	H5N1	2024AI05108	Germany-NI	10.12.2024	Meleagris gallopavo
**PV188009**	H5N1	2024AI05108	Germany-NI	10.12.2024	Meleagris gallopavo
**PV188010**	H5N1	2024AI05108	Germany-NI	10.12.2024	Meleagris gallopavo
**PV188011**	H5N1	2024AI05108	Germany-NI	10.12.2024	Meleagris gallopavo
**PV188012**	H5N1	2024AI05108	Germany-NI	10.12.2024	Meleagris gallopavo
**PV188013**	H5N1	2024AI05108	Germany-NI	10.12.2024	Meleagris gallopavo
**PV188014**	H5N1	2024AI05108	Germany-NI	10.12.2024	Meleagris gallopavo
**PV188015**	H5N1	2024AI05108	Germany-NI	10.12.2024	Meleagris gallopavo
**PQ736107**	H5N1	2024AI04198	Germany-ST	09.09.2024	Anser
**PQ736108**	H5N1	2024AI04198	Germany-ST	09.09.2024	Anser
**PQ736109**	H5N1	2024AI04198	Germany-ST	09.09.2024	Anser
**PQ736110**	H5N1	2024AI04198	Germany-ST	09.09.2024	Anser
**PQ736111**	H5N1	2024AI04198	Germany-ST	09.09.2024	Anser
**PQ736112**	H5N1	2024AI04198	Germany-ST	09.09.2024	Anser
**PQ736113**	H5N1	2024AI04198	Germany-ST	09.09.2024	Anser
**PQ736114**	H5N1	2024AI04198	Germany-ST	09.09.2024	Anser
**PQ736115**	H5N1	2024AI04219	Germany-BY	10.09.2024	Anser anser
**PQ736116**	H5N1	2024AI04219	Germany-BY	10.09.2024	Anser anser
**PQ736117**	H5N1	2024AI04219	Germany-BY	10.09.2024	Anser anser
**PQ736118**	H5N1	2024AI04219	Germany-BY	10.09.2024	Anser anser
**PQ736119**	H5N1	2024AI04219	Germany-BY	10.09.2024	Anser anser
**PQ736120**	H5N1	2024AI04219	Germany-BY	10.09.2024	Anser anser
**PQ736121**	H5N1	2024AI04219	Germany-BY	10.09.2024	Anser anser
**PQ736122**	H5N1	2024AI04219	Germany-BY	10.09.2024	Anser anser
**PQ736123**	H5N1	2024AI04242	Germany-HE	17.09.2024	Anatidae
**PQ736124**	H5N1	2024AI04242	Germany-HE	17.09.2024	Anatidae
**PQ736125**	H5N1	2024AI04242	Germany-HE	17.09.2024	Anatidae

**PQ736126**	H5N1	2024AI04242	Germany-HE	17.09.2024	Anatidae
**PQ736127**	H5N1	2024AI04242	Germany-HE	17.09.2024	Anatidae
**PQ736128**	H5N1	2024AI04271	Germany-MV	20.09.2024	Cygnus olor
**PQ736129**	H5N1	2024AI04271	Germany-MV	20.09.2024	Cygnus olor
**PQ736130**	H5N1	2024AI04271	Germany-MV	20.09.2024	Cygnus olor
**PQ736131**	H5N1	2024AI04271	Germany-MV	20.09.2024	Cygnus olor
**PQ736132**	H5N1	2024AI04271	Germany-MV	20.09.2024	Cygnus olor
**PQ736133**	H5N1	2024AI04271	Germany-MV	20.09.2024	Cygnus olor
**PQ736134**	H5N1	2024AI04271	Germany-MV	20.09.2024	Cygnus olor
**PQ736135**	H5N1	2024AI04271	Germany-MV	20.09.2024	Cygnus olor
**PQ736136**	H5N1	2024AI04274	Germany-MV	02.10.2024	Pelecanus
**PQ736137**	H5N1	2024AI04274	Germany-MV	02.10.2024	Pelecanus
**PQ736138**	H5N1	2024AI04274	Germany-MV	02.10.2024	Pelecanus
**PQ736139**	H5N1	2024AI04274	Germany-MV	02.10.2024	Pelecanus
**PQ736140**	H5N1	2024AI04274	Germany-MV	02.10.2024	Pelecanus
**PQ736141**	H5N1	2024AI04293	Germany-HH	08.10.2024	Anatidae
**PQ736142**	H5N1	2024AI04293	Germany-HH	08.10.2024	Anatidae
**PQ736143**	H5N1	2024AI04293	Germany-HH	08.10.2024	Anatidae
**PQ736144**	H5N1	2024AI04293	Germany-HH	08.10.2024	Anatidae
**PQ736145**	H5N1	2024AI04293	Germany-HH	08.10.2024	Anatidae
**PQ736146**	H5N1	2024AI04293	Germany-HH	08.10.2024	Anatidae
**PQ736147**	H5N1	2024AI04293	Germany-HH	08.10.2024	Anatidae
**PQ736148**	H5N1	2024AI04293	Germany-HH	08.10.2024	Anatidae
**PQ736149**	H5N1	2024AI04521	Germany-HH	18.10.2024	Cygnus olor
**PQ736150**	H5N1	2024AI04521	Germany-HH	18.10.2024	Cygnus olor
**PQ736151**	H5N1	2024AI04521	Germany-HH	18.10.2024	Cygnus olor
**PQ736152**	H5N1	2024AI04521	Germany-HH	18.10.2024	Cygnus olor
**PQ736153**	H5N1	2024AI04521	Germany-HH	18.10.2024	Cygnus olor
**PQ736154**	H5N1	2024AI04521	Germany-HH	18.10.2024	Cygnus olor
**PQ736155**	H5N1	2024AI04521	Germany-HH	18.10.2024	Cygnus olor
**PQ736156**	H5N1	2024AI04521	Germany-HH	18.10.2024	Cygnus olor
**PQ736157**	H5N1	2024AI04525	Germany-BY	24.10.2024	Anser
**PQ736158**	H5N1	2024AI04525	Germany-BY	24.10.2024	Anser
**PQ736159**	H5N1	2024AI04525	Germany-BY	24.10.2024	Anser
**PQ736160**	H5N1	2024AI04525	Germany-BY	24.10.2024	Anser
**PQ736161**	H5N1	2024AI04525	Germany-BY	24.10.2024	Anser
**PQ736162**	H5N1	2024AI04525	Germany-BY	24.10.2024	Anser
**PQ736163**	H5N1	2024AI04525	Germany-BY	24.10.2024	Anser
**PQ736164**	H5N1	2024AI04525	Germany-BY	24.10.2024	Anser
**PQ736165**	H5N1	2024AI04541	Germany-BY	22.10.2024	Branta canadensis
**PQ736166**	H5N1	2024AI04541	Germany-BY	22.10.2024	Branta canadensis
**PQ736167**	H5N1	2024AI04541	Germany-BY	22.10.2024	Branta canadensis
**PQ736168**	H5N1	2024AI04541	Germany-BY	22.10.2024	Branta canadensis
**PQ736169**	H5N1	2024AI04541	Germany-BY	22.10.2024	Branta canadensis
**PQ736170**	H5N1	2024AI04541	Germany-BY	22.10.2024	Branta canadensis

**PQ736171**	H5N1	2024AI04541	Germany-BY	22.10.2024	Branta canadensis
**PQ736172**	H5N1	2024AI04541	Germany-BY	22.10.2024	Branta canadensis
**PQ736173**	H5N1	2024AI04543	Germany-BY	28.10.2024	Anser
**PQ736174**	H5N1	2024AI04543	Germany-BY	28.10.2024	Anser
**PQ736175**	H5N1	2024AI04543	Germany-BY	28.10.2024	Anser
**PQ736176**	H5N1	2024AI04543	Germany-BY	28.10.2024	Anser
**PQ736177**	H5N1	2024AI04543	Germany-BY	28.10.2024	Anser
**PQ736178**	H5N1	2024AI04543	Germany-BY	28.10.2024	Anser
**PQ736179**	H5N1	2024AI04543	Germany-BY	28.10.2024	Anser
**PQ736180**	H5N1	2024AI04543	Germany-BY	28.10.2024	Anser
**PQ736181**	H5N1	2024AI04848	Germany-BY	07.11.2024	Astur gentilis
**PQ736182**	H5N1	2024AI04848	Germany-BY	07.11.2024	Astur gentilis
**PQ736183**	H5N1	2024AI04848	Germany-BY	07.11.2024	Astur gentilis
**PQ736184**	H5N1	2024AI04848	Germany-BY	07.11.2024	Astur gentilis
**PQ736185**	H5N1	2024AI04848	Germany-BY	07.11.2024	Astur gentilis
**PQ736186**	H5N1	2024AI04848	Germany-BY	07.11.2024	Astur gentilis
**PQ736187**	H5N1	2024AI04848	Germany-BY	07.11.2024	Astur gentilis
**PQ736188**	H5N1	2024AI04848	Germany-BY	07.11.2024	Astur gentilis
**PQ736189**	H5N1	2024AI04879	Germany-BY	16.11.2024	Anser
**PQ736190**	H5N1	2024AI04879	Germany-BY	16.11.2024	Anser
**PQ736191**	H5N1	2024AI04879	Germany-BY	16.11.2024	Anser
**PQ736192**	H5N1	2024AI04879	Germany-BY	16.11.2024	Anser
**PQ736193**	H5N1	2024AI04879	Germany-BY	16.11.2024	Anser
**PQ736194**	H5N1	2024AI04879	Germany-BY	16.11.2024	Anser
**PQ736195**	H5N1	2024AI04879	Germany-BY	16.11.2024	Anser
**PQ736196**	H5N1	2024AI04879	Germany-BY	16.11.2024	Anser
**PQ736197**	H5N1	2024AI04292	Germany-TH	07.10.2024	Branta canadensis
**PQ736198**	H5N1	2024AI04292	Germany-TH	07.10.2024	Branta canadensis
**PQ736199**	H5N1	2024AI04292	Germany-TH	07.10.2024	Branta canadensis
**PQ736200**	H5N1	2024AI04292	Germany-TH	07.10.2024	Branta canadensis
**PQ736201**	H5N1	2024AI04292	Germany-TH	07.10.2024	Branta canadensis
**PQ736202**	H5N1	2024AI04292	Germany-TH	07.10.2024	Branta canadensis
**PQ736203**	H5N1	2024AI04292	Germany-TH	07.10.2024	Branta canadensis
**PQ736204**	H5N1	2024AI04292	Germany-TH	07.10.2024	Branta canadensis
**PQ736205**	H5N1	2024AI04295	Germany-SH	08.10.2024	Larus marinus
**PQ736206**	H5N1	2024AI04295	Germany-SH	08.10.2024	Larus marinus
**PQ736207**	H5N1	2024AI04295	Germany-SH	08.10.2024	Larus marinus
**PQ736208**	H5N1	2024AI04295	Germany-SH	08.10.2024	Larus marinus
**PQ736209**	H5N1	2024AI04295	Germany-SH	08.10.2024	Larus marinus
**PQ736210**	H5N1	2024AI04295	Germany-SH	08.10.2024	Larus marinus
**PQ736211**	H5N1	2024AI04295	Germany-SH	08.10.2024	Larus marinus
**PQ736212**	H5N1	2024AI04295	Germany-SH	08.10.2024	Larus marinus
**PQ736213**	H5N1	2024AI03518	Germany-MV	19.07.2024	Cygnus olor
**PQ736214**	H5N1	2024AI03518	Germany-MV	19.07.2024	Cygnus olor
**PQ736215**	H5N1	2024AI03518	Germany-MV	19.07.2024	Cygnus olor

**PQ736216**	H5N1	2024AI03518	Germany-MV	19.07.2024	Cygnus olor
**PQ736217**	H5N1	2024AI03518	Germany-MV	19.07.2024	Cygnus olor
**PQ736218**	H5N1	2024AI03518	Germany-MV	19.07.2024	Cygnus olor
**PQ736219**	H5N1	2024AI03518	Germany-MV	19.07.2024	Cygnus olor
**PQ736220**	H5N1	2024AI03518	Germany-MV	19.07.2024	Cygnus olor
**PQ736221**	H5N1	2024AI04248	Germany-MV	19.09.2024	Cygnus olor
**PQ736222**	H5N1	2024AI04248	Germany-MV	19.09.2024	Cygnus olor
**PQ736223**	H5N1	2024AI04248	Germany-MV	19.09.2024	Cygnus olor
**PQ736224**	H5N1	2024AI04248	Germany-MV	19.09.2024	Cygnus olor
**PQ736225**	H5N1	2024AI04248	Germany-MV	19.09.2024	Cygnus olor
**PQ736226**	H5N1	2024AI04248	Germany-MV	19.09.2024	Cygnus olor
**PQ736227**	H5N1	2024AI04248	Germany-MV	19.09.2024	Cygnus olor
**PQ736228**	H5N1	2024AI04248	Germany-MV	19.09.2024	Cygnus olor
**PQ736229**	H5N1	2024AI04155	Germany-MV	12.08.2024	Ciconia ciconia
**PQ736230**	H5N1	2024AI04155	Germany-MV	12.08.2024	Ciconia ciconia
**PQ736231**	H5N1	2024AI04155	Germany-MV	12.08.2024	Ciconia ciconia
**PQ736232**	H5N1	2024AI04155	Germany-MV	12.08.2024	Ciconia ciconia
**PQ736233**	H5N1	2024AI04155	Germany-MV	12.08.2024	Ciconia ciconia
**PQ736234**	H5N1	2024AI04155	Germany-MV	12.08.2024	Ciconia ciconia
**PQ736235**	H5N1	2024AI04155	Germany-MV	12.08.2024	Ciconia ciconia
**PQ736236**	H5N1	2024AI04155	Germany-MV	12.08.2024	Ciconia ciconia
**PQ736237**	H5N1	2024AI05008	Germany-SH	22.11.2024	Gallus gallus
**PQ736238**	H5N1	2024AI05008	Germany-SH	22.11.2024	Gallus gallus
**PQ736239**	H5N1	2024AI05008	Germany-SH	22.11.2024	Gallus gallus
**PQ736240**	H5N1	2024AI05008	Germany-SH	22.11.2024	Gallus gallus
**PQ736241**	H5N1	2024AI05008	Germany-SH	22.11.2024	Gallus gallus
**PQ736242**	H5N1	2024AI05008	Germany-SH	22.11.2024	Gallus gallus
**PQ736243**	H5N1	2024AI05008	Germany-SH	22.11.2024	Gallus gallus
**PQ736244**	H5N1	2024AI05008	Germany-SH	22.11.2024	Gallus gallus
**PQ736245**	H5N1	2024AI04001	Germany-MV	11.08.2024	Anas platyrhynchos
**PQ736246**	H5N1	2024AI04001	Germany-MV	11.08.2024	Anas platyrhynchos
**PQ736247**	H5N1	2024AI04001	Germany-MV	11.08.2024	Anas platyrhynchos
**PQ736248**	H5N1	2024AI04001	Germany-MV	11.08.2024	Anas platyrhynchos
**PQ736249**	H5N1	2024AI04001	Germany-MV	11.08.2024	Anas platyrhynchos
**PQ736250**	H5N1	2024AI04001	Germany-MV	11.08.2024	Anas platyrhynchos
**PQ736251**	H5N1	2024AI04001	Germany-MV	11.08.2024	Anas platyrhynchos
**PQ736252**	H5N1	2024AI04001	Germany-MV	11.08.2024	Anas platyrhynchos
**PQ736253**	H5N1	2024AI04250	Germany-SN	26.09.2024	Anser
**PQ736254**	H5N1	2024AI04250	Germany-SN	26.09.2024	Anser
**PQ736255**	H5N1	2024AI04250	Germany-SN	26.09.2024	Anser
**PQ736256**	H5N1	2024AI04250	Germany-SN	26.09.2024	Anser
**PQ736257**	H5N1	2024AI04250	Germany-SN	26.09.2024	Anser
**PQ736258**	H5N1	2024AI04250	Germany-SN	26.09.2024	Anser
**PQ736259**	H5N1	2024AI04250	Germany-SN	26.09.2024	Anser
**PQ736260**	H5N1	2024AI04250	Germany-SN	26.09.2024	Anser

**PQ736261**	H5N1	2024AI04832	Germany-BY	04.11.2024	Anatidae
**PQ736262**	H5N1	2024AI04832	Germany-BY	04.11.2024	Anatidae
**PQ736263**	H5N1	2024AI04832	Germany-BY	04.11.2024	Anatidae
**PQ736264**	H5N1	2024AI04832	Germany-BY	04.11.2024	Anatidae
**PQ736265**	H5N1	2024AI04832	Germany-BY	04.11.2024	Anatidae
**PQ736266**	H5N1	2024AI04832	Germany-BY	04.11.2024	Anatidae
**PQ736267**	H5N1	2024AI04832	Germany-BY	04.11.2024	Anatidae
**PQ736268**	H5N1	2024AI04832	Germany-BY	04.11.2024	Anatidae
**PQ736269**	H5N1	2024AI04220	Germany-HE	13.09.2024	Anatidae
**PQ736270**	H5N1	2024AI04220	Germany-HE	13.09.2024	Anatidae
**PQ736271**	H5N1	2024AI04220	Germany-HE	13.09.2024	Anatidae
**PQ736272**	H5N1	2024AI04220	Germany-HE	13.09.2024	Anatidae
**PQ736273**	H5N1	2024AI04220	Germany-HE	13.09.2024	Anatidae
**PQ736274**	H5N1	2024AI04220	Germany-HE	13.09.2024	Anatidae
**PQ736275**	H5N1	2024AI04220	Germany-HE	13.09.2024	Anatidae
**PQ736276**	H5N1	2024AI04220	Germany-HE	13.09.2024	Anatidae
**PQ736277**	H5N1	2024AI04224	Germany-HH	17.09.2024	Anatidae
**PQ736278**	H5N1	2024AI04224	Germany-HH	17.09.2024	Anatidae
**PQ736279**	H5N1	2024AI04224	Germany-HH	17.09.2024	Anatidae
**PQ736280**	H5N1	2024AI04224	Germany-HH	17.09.2024	Anatidae
**PQ736281**	H5N1	2024AI04224	Germany-HH	17.09.2024	Anatidae
**PQ736282**	H5N1	2024AI04224	Germany-HH	17.09.2024	Anatidae
**PQ736283**	H5N1	2024AI04224	Germany-HH	17.09.2024	Anatidae
**PQ736284**	H5N1	2024AI04224	Germany-HH	17.09.2024	Anatidae
**PQ736285**	H5N1	2024AI04875	Germany-NI	06.11.2024	Anatidae
**PQ736286**	H5N1	2024AI04875	Germany-NI	06.11.2024	Anatidae
**PQ736287**	H5N1	2024AI04875	Germany-NI	06.11.2024	Anatidae
**PQ736288**	H5N1	2024AI04875	Germany-NI	06.11.2024	Anatidae
**PQ736289**	H5N1	2024AI04875	Germany-NI	06.11.2024	Anatidae
**PQ736290**	H5N1	2024AI04875	Germany-NI	06.11.2024	Anatidae
**PQ736291**	H5N1	2024AI04875	Germany-NI	06.11.2024	Anatidae
**PQ736292**	H5N1	2024AI04875	Germany-NI	06.11.2024	Anatidae
**PQ736293**	H5N1	2024AI04233	Germany-ST	11.09.2024	Anatidae
**PQ736294**	H5N1	2024AI04233	Germany-ST	11.09.2024	Anatidae
**PQ736295**	H5N1	2024AI04233	Germany-ST	11.09.2024	Anatidae
**PQ736296**	H5N1	2024AI04233	Germany-ST	11.09.2024	Anatidae
**PQ736297**	H5N1	2024AI04233	Germany-ST	11.09.2024	Anatidae
**PQ736298**	H5N1	2024AI04233	Germany-ST	11.09.2024	Anatidae
**PQ736299**	H5N1	2024AI04233	Germany-ST	11.09.2024	Anatidae
**PQ736300**	H5N1	2024AI04233	Germany-ST	11.09.2024	Anatidae
**PQ736301**	H5N1	2024AI05010	Germany-NI	23.11.2024	Meleagris gallopavo
**PQ736302**	H5N1	2024AI05010	Germany-NI	23.11.2024	Meleagris gallopavo
**PQ736303**	H5N1	2024AI05010	Germany-NI	23.11.2024	Meleagris gallopavo
**PQ736304**	H5N1	2024AI05010	Germany-NI	23.11.2024	Meleagris gallopavo
**PQ736305**	H5N1	2024AI05010	Germany-NI	23.11.2024	Meleagris gallopavo

**PQ736306**	H5N1	2024AI05010	Germany-NI	23.11.2024	Meleagris gallopavo
**PQ736307**	H5N1	2024AI05010	Germany-NI	23.11.2024	Meleagris gallopavo
**PQ736308**	H5N1	2024AI05010	Germany-NI	23.11.2024	Meleagris gallopavo
**PQ736309**	H5N1	2024AI04873	Germany-NW	17.11.2024	Meleagris gallopavo
**PQ736310**	H5N1	2024AI04873	Germany-NW	17.11.2024	Meleagris gallopavo
**PQ736311**	H5N1	2024AI04873	Germany-NW	17.11.2024	Meleagris gallopavo
**PQ736312**	H5N1	2024AI04873	Germany-NW	17.11.2024	Meleagris gallopavo
**PQ736313**	H5N1	2024AI04873	Germany-NW	17.11.2024	Meleagris gallopavo
**PQ736314**	H5N1	2024AI04873	Germany-NW	17.11.2024	Meleagris gallopavo
**PQ736315**	H5N1	2024AI04873	Germany-NW	17.11.2024	Meleagris gallopavo
**PQ736316**	H5N1	2024AI04873	Germany-NW	17.11.2024	Meleagris gallopavo
**PQ727365**	H5N5	2024AI04273	Germany-NI	21.09.2024	Fulmarus glacialis
**PQ727366**	H5N5	2024AI04273	Germany-NI	21.09.2024	Fulmarus glacialis
**PQ727367**	H5N5	2024AI04273	Germany-NI	21.09.2024	Fulmarus glacialis
**PQ727368**	H5N5	2024AI04273	Germany-NI	21.09.2024	Fulmarus glacialis
**PQ727369**	H5N5	2024AI04273	Germany-NI	21.09.2024	Fulmarus glacialis
**PQ727370**	H5N5	2024AI04273	Germany-NI	21.09.2024	Fulmarus glacialis
**PQ727371**	H5N5	2024AI04273	Germany-NI	21.09.2024	Fulmarus glacialis
**PQ727372**	H5N5	2024AI04273	Germany-NI	21.09.2024	Fulmarus glacialis

Table S2 Overview of sequence counts per country and per genotype for datasets DI.1, DI.2, EF, and EE included in the BEAST analyses for evaluation of potential sampling bias.

country	**DI.1**	**DI.2**	**EE**	**EF**
Albania		3		
Austria	5	25	1	
Belgium		5		
Bulgaria		1		
Croatia	7	5		
Czech Republic	25	39		
Denmark	1	4	1	1
England	3	13		
Finland		3		
France		1		
Germany	15	37	3	6
Ireland		2		
Italy	4	28		
Moldova	7			
Netherlands	2	6		8
Norway				1
Poland	15	11		
Romania	13	3		
Serbia		1		
Slovakia	11	3		
Slovenia		19		
Sweden		1	1	3

Table S3 Estimates of time to the most recent common ancestor (tMRCA) and substitution rates inferred using BEAST for datasets DI.1, DI.2, EF, and EE. For each dataset, the mean, 95% highest posterior density (HPD) interval, and effective sample size (ESS) are reported. Substitution rates are given in substitutions per site per year. All analyses were performed using a Bayesian MCMC framework with the indicated chain lengths and a burn-in fraction of 10%. ESS values (>200) indicate adequate sampling of the posterior distribution.

	**DI.1**	**DI.2**	**EF**	**EE**
**MCMC chain length**	100000000	100000000	20000000	30000000
**Burn in fraction**	0.1	0.1	0.1	0.1
**tMRCA (mean)**	2023.68	2023.98	2024.76	2022.48
**tMRCA (95% HPD)**	[2023.517, 2023.7943]	[2023.6895, 2024.2387]	[2024.6238, 2024.8587]	[2014.3244, 2025.0126]
**tMRCA (ESS)**	547	534	239	826
**Substitution rate (mean)**	7.61E-03	5.11E-03	4.63E-03	3.50E-03
**Substitution rate (95% HPD)**	[6.034E-3, 9.2379E-3]	[4.4959E-3, 5.7132E-3]	[3.4973E-3, 5.8272E-3]	[6.3023E-6, 0.0105]
**Substitution rate (ESS)**	474.5	217	812	485
